# Immunogenicity of a single-dose compared with a two-dose primary series followed by a booster dose of ten-valent or 13-valent pneumococcal conjugate vaccine in South African children: an open-label, randomised, non-inferiority trial

**DOI:** 10.1016/S1473-3099(20)30289-9

**Published:** 2020-12

**Authors:** Shabir A Madhi, Eleonora AML Mutsaerts, Alane Izu, Welekazi Boyce, Sutika Bhikha, Benit T Ikulinda, Lisa Jose, Anthonet Koen, Amit J Nana, Andrew Moultrie, Lucy Roalfe, Adam Hunt, David Goldblatt, Clare L Cutland, Jeffrey R Dorfman

**Affiliations:** a South African Medical Research Council Vaccines and Infectious Diseases Analytical Research Unit, University of the Witwatersrand, Faculty of Health Science, Johannesburg, South Africa; b Department of Science, National Research Foundation: Vaccine Preventable Diseases, University of the Witwatersrand, Faculty of Health Science, Johannesburg, South Africa; c Immunobiology Section, University College London, Great Ormond Street Institute of Child Health Biomedical Research Centre, London, UK

## Abstract

**Background:**

Routine childhood immunisation with pneumococcal conjugate vaccine (PCV) has changed the epidemiology of pneumococcal disease across age groups, providing an opportunity to reconsider PCV dosing schedules. We aimed to evaluate the post-booster dose immunogenicity of ten-valent (PCV10) and 13-valent (PCV13) PCVs between infants randomly assigned to receive a single-dose compared with a two-dose primary series.

**Methods:**

We did an open-label, non-inferiority, randomised study in HIV-unexposed infants at a single centre in Soweto, South Africa. Infants were randomly assigned to receive one priming dose of PCV10 or PCV13 at ages 6 weeks (6w + 1 PCV10 and 6w + 1 PCV13 groups) or 14 weeks (14w + 1 PCV10 and 14w + 1 PCV13 groups) or two priming doses of PCV10 or PCV13, one each at ages 6 weeks and 14 weeks (2 + 1 PCV10 and 2 + 1 PCV13 groups); all participants then received a booster dose of PCV10 or PCV13 at 40 weeks of age. The primary endpoint was geometric mean concentrations (GMCs) of serotype-specific IgG 1 month after the booster dose, which was assessed in all participants who received PCV10 or PCV13 as per the assigned randomisation group and for whom laboratory results were available at that timepoint. The 1 + 1 vaccine schedule was considered non-inferior to the 2 + 1 vaccine schedule if the lower bound of the 96% CI for the GMC ratio was greater than 0·5 for at least ten PCV13 serotypes and eight PCV10 serotypes. Safety was a secondary endpoint. This trial is registered with ClinicalTrials.gov (NCT02943902) and is ongoing.

**Findings:**

Of 1695 children assessed, 600 were enrolled and randomly assigned to one of the six groups between Jan 9 and Sept 20, 2017; 542 were included in the final analysis of the primary endpoint (86–93 per group). For both PCV13 and PCV10, a 1+1 dosing schedule (either beginning at 6 or 14 weeks) was non-inferior to a 2 + 1 schedule. For PCV13, the lower limit of the 96% CI for the ratio of GMCs between the 1 + 1 and 2 + 1 groups was higher than 0·5 for ten serotypes in the 6w+1 group (excluding 6B, 14, and 23F) and 11 serotypes in the 14w + 1 group (excluding 6B and 23F). For PCV10, the lower limit of the 96% CI for the ratio of GMCs was higher than 0·5 for all ten serotypes in the 6w+1 and 14w + 1 groups. 84 serious adverse events were reported in 72 (12%) of 600 participants. 15 occurred within 28 days of vaccination, but none were considered to be related to PCV injection. There were no cases of culture-confirmed invasive pneumococcal disease.

**Interpretation:**

The non-inferiority in post-booster immune responses following a single-dose compared with a two-dose primary series of PCV13 or PCV10 indicates the potential for reducing PCV dosing schedules from a 2 + 1 to 1 + 1 series in low-income and middle-income settings with well established PCV immunisation programmes.

**Funding:**

The Bill & Melinda Gates Foundation (OPP1 + 152352).

## Introduction

WHO recommends immunisation of children with ten-valent (PCV10) or 13-valent (PCV13) pneumococcal conjugate vaccine (PCV), with either three doses given during early infancy or two doses given in early infancy and a booster dose given from age 9 months onward (so-called 2 + 1 schedule).^1^ Although both PCV dosing schedules and valencies are effective in preventing invasive pneumococcal disease caused by vaccine serotypes,^2^ the absence of a booster dose has been associated with waning immunity.^3^, ^4^, ^5^

As well as preventing vaccine-type disease, PCV immunisation of infants also reduces the risk of nasopharyngeal acquisition of *Streptococcus pneumoniae* serotypes included in the vaccine.^6^ Although a correlate of protection against pneumococcal colonisation has not been definitively established, a meta-analysis of PCV10-related studies observed an inverse association between serotype-specific IgG and sero-epidemiological evidence of colonisation by the homotypic serotype.^7^ Also, the serotype-specific IgG concentration estimated to protect against colonisation was higher than that required to protect against invasive pneumococcal disease.^7^ Children aged between 1 year and 4 years are considered the main source of pneumococcal transmission.^8^, ^9^, ^10^ Transmission of pneumococci is predominantly from children to adults, even in settings with a high prevalence of HIV.^11^ Hence, the effectiveness of routine childhood PCV immunisation in reducing transmission of vaccine serotypes in the community might be affected by eliciting or sustaining high IgG concentrations in children aged 1–4 years, which could be optimised with a booster dose of PCV.

**Research in context**

**Evidence before this study**

Routine immunisation of children with pneumococcal conjugate vaccine (PCV) has resulted in major changes in the epidemiology of pneumococcal disease among the age group targeted for vaccination, as well as among those not targeted (through an indirect effect). Furthermore, there has been near elimination of colonisation by and transmission of vaccine-type pneumococci in settings that include PCV in their routine childhood immunisation programmes. Consequently, repurposing the focus of childhood PCV immunisation to sustain the effect of immunisation on vaccine-serotype colonisation is being considered, including the possibility of reducing the number of doses of PCV in the primary series schedule which could also reduce the cost of PCV immunisation. We searched PubMed up to Jan 31, 2020, for clinical trials that evaluated immunogenicity following a booster dose of PCV in children primed with a single-dose versus a two-dose primary series of ten-valent (PCV10) or 13-valent PCV (PCV13). We used various combinations of the search terms “pneumococcal conjugate vaccine”, “pneumococcal vaccine”, ”immunogenicity”, ”dosing schedule”, ”meta-analysis”, ”systematic review”, and ”randomized controlled trial”. We identified a single previous study on PCV13 from the UK, which reported that serotype-specific IgG geometric mean concentrations (GMCs) following a booster dose of PCV13 at age 12 months were non-inferior in children who received a single priming (1 + 1) dose at age 3 months compared with those vaccinated with a two-dose primary series at ages 2 and 4 months (2 + 1). The study concluded that post-booster immune responses in infants primed with a single dose of PCV13 were equivalent or superior (for serotypes 1, 4, 14, and 19F) to those primed with two doses of PCV, except for serotypes 6B and 23F.

**Added value of this study**

We did an open-label randomised controlled trial to evaluate whether post-booster serotype-specific GMCs in children randomised to receive either PCV10 or PCV13 as a 1 + 1 schedule (with the first dose occurring either at 6 or 14 weeks of age) were non-inferior to those in infants who received a two-dose primary series (at 6 and 14 weeks of age). All six study groups received a booster dose at 40 weeks of age, and serotype-specific IgG and opsonophagocytic activity (in a subset) were measured 1 month post-booster (as well as 1 month after the respective primary series and immediately before the booster dose of vaccine). For PCV13, our data corroborate the data from the UK (albeit with vaccination at different timepoints) of the non-inferiority of post-booster GMCs in infants vaccinated with a 1 + 1 compared with a 2 + 1 schedule, with the 1 + 1 groups actually having higher GMCs for at least five serotypes (1, 4, 9V, 19A, and 19F) in our study. Also, to our knowledge, we provide the first evidence for non-inferiority in post-booster GMCs following a single compared with a two-dose primary series of PCV10. The post-booster opsonophagocytic activity (functional antibody) also did not differ substantially between the 1+1 and 2 + 1 schedules for PCV13 or PCV10. Furthermore, for PCV13 (and for serotype 18C in PCV10, which is the only serotype conjugated to diphtheria toxoid), we show that delaying the first dose in the 1 + 1 schedule to 14 weeks (*vs* 6 weeks) was associated with higher post-first dose GMCs and a higher percentage of children with serotype-specific antibody concentrations above the putative correlate of protection for invasive pneumococcal diseases. This enhanced immunogenicity persisted up to 9 months of age when the booster dose was given.

**Implications of all the available evidence**

We provide corroborating immunological evidence for the case of transitioning from a 2 + 1 to a 1 + 1 dosing schedule of PCV13 or PCV10, including in settings where the booster dose is given as early as 40 weeks of age. More data on the epidemiology of pneumococcal colonisation and disease are, however, needed from our setting and other similar low-income and middle-income settings with established PCV childhood immunisation programmes. This information, coupled with a cost, benefit, and risk assessment, and capabilities to ensure high rates of coverage with a 1 + 1 dosing schedule, would enable deliberations on whether countries such as South Africa should also consider transitioning to a 1 + 1 PCV dosing schedule as has been done in the UK since 2020. The motivation for the reduced PCV dosing schedule in settings such as ours include decreasing the number of injectable vaccines being administered to infants and reducing the cost of PCV procurement, which is a major impediment to the introduction of PCV into public immunisation programmes for many middle-income countries.

Routine and widespread PCV immunisation of children have led to near elimination of invasive pneumococcal disease caused by vaccine serotypes in high-income and low-income and middle-income settings, including in age groups not targeted for vaccination.^12^ In South Africa, following the introduction of routine PCV immunisation of infants in 2009, the incidence of vaccine-serotype invasive pneumococcal disease declined by more than 90% in the child age group targeted for vaccination, and by more than 85% in age groups not targeted for PCV immunisation.^13^ The public health benefit of the indirect (herd) effect of childhood PCV immunisation exceeds the direct protection conferred to vaccinated children in settings with well established childhood PCV immunisation programmes.^12^ In such settings, the focus of infant PCV immunisation could be repurposed to sustain the effectiveness of immunisation on maintaining low rates of vaccine-serotype colonisation (and by proxy, disease) at a population level.^14^

The UK transitioned from a 2 + 1 (immunisation at 2, 4, and 12 months of age) to a 1 + 1 (immunisation at 12 weeks and 12–13 months of age) childhood PCV13 dosing schedule in 2020.^15^ The effects of this transition have yet to be assessed. Further studies of the immunogenicity of the PCV 1+1 dosing schedule are needed, especially in low-income and middle-income countries, and also in relation to PCV10, as extrapolating from the UK study to different populations has its limitations. Furthermore, the effect of timing of the first PCV dose on immunogenicity when used in a 1 + 1 PCV schedule needs to be investigated, especially for countries in which vaccination is given at a younger age, such as South Africa where the primary series of the existing 2 + 1 schedule is at 6 and 14 weeks of age.

The aim of this study was to evaluate the immunogenicity and safety of a 1 + 1 PCV schedule comprising a single priming dose of PCV10 or PCV13 at 6 or 14 weeks of age, followed by a booster dose at 40 weeks of age, compared with a 2 + 1 PCV schedule with doses given at 6, 14, and 40 weeks of age.

## Methods

### Study design and participants

A single-centre, open-label, non-inferiority, randomised trial, was done in Soweto, South Africa, with enrolment from Jan 9 to Sept 20, 2017. We included healthy infants aged 42–56 days who were born to HIV-uninfected women and had not received any vaccines (except for BCG and oral polio at birth). Other inclusion and exclusion criteria are described in the appendix (p 1). Screening for study participants was done at neighbouring immunisation clinics or following delivery at Chris Hani Baragwanath Academic Hospital (CHBAH), Soweto, South Africa. Infants who were potentially eligible and whose parent(s) indicated willingness for their children to participate were referred to the Respiratory and Meningeal Pathogens Research Unit (RMPRU, Johannesburg, South Africa) based at CHBAH for enrolment. All subsequent study visits and vaccinations of enrolled participants were done at RMPRU. Written informed consent was obtained from parents before enrolment of their children.

The protocol was approved by the Human Research Ethics Committee, University of the Witwatersrand, Johannesburg, South Africa, and the South African Health Products Regulatory Authority. The full study protocol is available online.

### Randomisation and masking

Infants were randomly assigned (1:1:1:1:1:1) through block randomisation (block size 30) to one of the six study groups. Study numbers and the corresponding randomisation group were allocated to eligible participants in sequential order by study staff. Randomisation allocated infants to receive a single priming dose of PCV10 (Synflorix, GlaxoSmithKline, Rixensart, Belgium; which includes serotypes 1, 4, 5, 6B, 7F, 9V, 14, 18C, 19FD and 23F) or PCV13 (Prevnar-13, Pfizer, New York City, USA; which includes all PCV10 serotypes and serotypes 3, 6A, and 19A) at 6 weeks (6w + 1 PCV10 group or 6w + 1 PCV13 group) or 14 weeks (14w + 1 PCV10 group or 14w + 1 PCV13 group) of age or two priming doses, one each at 6 and 14 weeks of age (2 + 1 PCV10 group or 2 + 1 PCV13 group). Infants in all groups received a booster dose of the corresponding PCV formulation at 40 weeks of age. Parents of participants and clinical staff were not masked to study-group assignment. Laboratory personnel were masked to the participant's identity and randomisation assignment throughout the study.

### Procedures

Infants received either two or three intramuscular injections (0·5 mL) of PCV10 or PCV13 in the anterolateral thigh at the vaccination visits. Details of other concomitantly administered vaccines are described in the appendix (p 1). In addition to collection of clotted blood for PCV serology analyses 1 month after the booster dose in all study groups, blood was collected 1 month following completion of the allocated primary series of PCV and immediately before the booster dose (at 40 weeks of age; appendix p 1).

Clotted blood samples were labelled with a unique laboratory number and delivered to the RMPRU laboratory within 4 h of collection, where samples were centrifuged and sera stored at −70°C. Serotype-specific IgG concentrations were measured for all PCV13 capsular polysaccharides by use of an in-house ELISA according to the standardised WHO protocol,^16^ and by use of the 007sp WHO standard for determination of serotype-specific antibody concentrations,^17^ as detailed in the appendix (pp 1–2). Opsonophagocytic activity (OPA) was tested 1 month after the booster dose in a subset of participants (20 per group) at the WHO pneumococcal serology reference laboratory (Great Ormond Street Institute of Child Health, University College London, London, UK).^18^ These participants were randomly selected by means of a random number generator with study group as a blocking factor.

Passive surveillance was done throughout the study for adverse events and serious adverse events, including pneumonia and invasive pneumococcal disease.

### Outcomes

The primary endpoint was serotype-specific IgG geometric mean concentrations (GMCs) 1 month following the booster dose (of PCV10 or PCV13) in the 1 + 1 PCV groups compared with the 2 + 1 PCV groups.

Secondary endpoints included GMCs and proportions of participants with vaccine serotype-specific serum IgG antibody concentrations above the putative correlate of protection for invasive pneumococcal disease (≥0·35 μg/mL;^19^ ie, seroprotection). These endpoints were evaluated 1 month after the respective one-dose or two-dose primary series, immediately before the booster dose (at 40 weeks age), and 1 month after the booster dose. Although comparison of post-booster OPA among study groups was also a prespecified secondary endpoint, considering that OPA testing was only done in 20 per group (owing to resource constraints), we did not do any statistical tests between study groups for this endpoint and report only descriptive statistics.

Secondary safety outcomes included the number of participants reporting serious adverse events in the 1 + 1 and 2 + 1 dosing groups throughout the study. Details of other secondary objectives and the future reporting thereof are detailed in the appendix (p 2).

### Statistical analysis

The study sample size was designed to have at least 80% power to establish non-inferiority for ten of the PCV13 and eight of the PCV10 serotypes. Assuming an SD for the log antibody concentrations of 0·4, the true ratio of GMCs between 1 + 1 and 2 + 1 groups being 1·05, and a type I error of 0·05, 91 evaluable participants per group post-booster dose were required. The sample size was upwardly adjusted to 100 per group to accommodate possible non-evaluable participants.

GMCs and 95% CIs are reported for serotype-specific antibody measurements for each group 1 month following completion of the primary series, immediately before the booster dose, and at 1 month after the booster dose. Non-inferiority was shown if the lower limit of the 96% CI of the ratio of GMCs between the 1 + 1 and 2 + 1 dosing schedules was greater than 0·5 for at least ten of the PCV13 and eight of the PCV10 serotypes. A 96% CI was used to control the overall type I error.^20^ 96% CIs for the ratios of GMCs between the 1 + 1 and 2 + 1 groups were calculated by back transformation of the CIs for the means of the log-transformed antibody concentrations.

GMCs were said to be higher in the 1 + 1 group than in the 2 + 1 group if the lower limit of the 96% CI for the ratio of GMCs was greater than 1, and lower in the 1 + 1 group than in the 2 + 1 group if the upper limit of the 96% CI for the ratio of GMCs was less than 1 for all GMC comparisons. GMCs between groups were also compared by means of a Student's *t* test.

Proportions of participants with putative sero-correlate of protection against invasive pneumococcal disease were calculated and expressed as point estimates with CIs; 96% CIs were calculated for the difference in proportions between groups. Non-inferiority criteria for seroprotection were met if the lower bound of the 96% CI for the difference in percentage between the 1 + 1 group and the 2 + 1 group was −10% or above for at least ten of the PCV13 serotypes and eight of the PCV10 serotypes. A Z test was also used to compare seroprotection between the 1 + 1 and 2 + 1 groups with a two-sided alpha level of 0·05.

All analyses used the modified intention-to-treat population, which included all participants who received PCV10 or PCV13 as per the assigned randomisation group and for whom laboratory results were available at all timepoints of blood sampling. A Data Safety and Monitoring Board monitored safety data pooled by the randomisation groups. All analyses were done in R version 3.5. This study is registered with ClinicalTrials.gov, NCT02943902.

### Role of the funding source

The funder of the study had no role in study design, data collection, data analysis, data interpretation, or writing of the report. The corresponding author had full access to all the data in the study and final responsibility for the decision to submit for publication.

## Results

**Figure 1:**
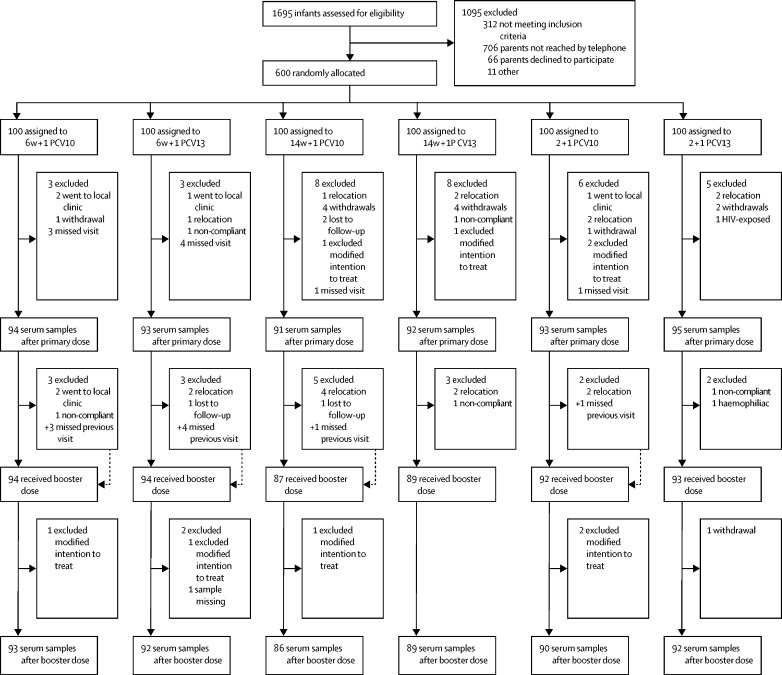
Trial profile Participants reincluded are indicated with a + symbol. Infants were randomly assigned to receive one primary dose of PCV10 or PCV13 at age 6 weeks (6w + 1 PCV10 and PCV13 groups) or 14 weeks (14w + 1 PCV10 and PCV13 groups) or two primary doses, one each at ages 6 weeks and 14 weeks (2 + 1 groups). All infants received a booster dose at age 40 weeks. PCV10=ten-valent pneumococcal conjugate vaccine. PCV13=13-valent pneumococcal conjugate vaccine.

Of 1695 infants assessed for eligibility between Jan 9 and Sept 20, 2017, 600 infants were enrolled and randomly assigned to one of six study groups (figure 1). 593 (99%) infants were black African, 310 (52%) were male, and the mean weight-for-age Z score was 0·6 (SD 0·96) at enrolment. Age at vaccination and blood sampling post-booster and other demographic characteristics were similar across the study groups (table).

**Table:** Demographics of study population

		**6w + 1 PCV10 (n=100)**	**6w + 1 PCV13 (n=100)**	**14w + 1 PCV10 (n=100)**	**14w + 1 PCV13 (n=100)**	**2 + 1 PCV10 (n=100)**	**2 + 1 PCV13 (n=100)**
Sex							
	Female	45 (45%)	51 (51%)	52 (52%)	55 (55%)	45 (45%)	42 (42%)
	Male	55 (55%)	49 (49%)	48 (48%)	45 (45%)	55 (55%)	58 (58%)
Race							
	Black African	100 (100%)	98 (98%)	100 (100%)	99 (99%)	97 (97%)	99 (99%)
	Mixed	0	2 (2%)	0	1 (1%)	2 (2%)	1 (1%)
Birthweight, g		3262·3 (431·6)	3238·5 (467·2)	3232·1 (392·1)	3203·6 (433·6)	3308·1 (478·2)	3177·8 (400·6)
Weight at enrolment, kg		4·93 (0·76)	4·92 (0·65)	4·89 (0·55)	4·83 (0·58)	4·95 (0·73)	4·79 (0·61)
Weight-for-age Z score at enrolment		0·09 (1·06)	0·13 (0·95)	0·12 (0·85)	0·01 (0·85)	0·12 (1·08)	0·11 (0·94)
Age at first PCV dose, weeks		6·39 (0·42)	6·36 (0·42)	14·43 (0·53)	14·58 (0·65)	6·42 (0·41)	6·37 (0·4)
Age at second PCV primary dose, weeks		NA	NA	NA	NA	15·08 (5·31)	14·5 (0·63)
Age at booster dose, months		8·96 (0·15)	8·98 (0·17)	9·03 (0·4)	8·98 (0·09)	9·02 (0·39)	9·03 (0·52)
Number sampled before booster vaccine		76 (76%)	73 (73%)	69 (69%)	69 (69%)	75 (75%)	76 (76%)
Number receiving booster dose		94 (94%)	94 (94%)	88 (88%)	90 (90%)	94 (94%)	93 (93%)
Weight-for-age Z score at booster dose*		0·23 (1·32)	0·26 (1·21)	−0·02 (1·12)	−0·13 (1·13)	0·14 (1·41)	−0·07 (1·28)
Number sampled 1 month post-booster dose		94 (94%)	93 (93%)	87 (87%)	90 (90%)	94 (94%)	92 (92%)
Age at post-booster dose sampling, months		9·93 (0·16)	9·94 (0·14)	9·99 (0·42)	9·95 (0·15)	9·95 (0·19)	10 (0·53)

Data are n (%) or mean (SD). Infants were randomly assigned to receive one primary dose of PCV10 or PCV13 at age 6 weeks (6w + 1 PCV10 and PCV13 groups) or 14 weeks (14w + 1 PCV10 and PCV13 groups) or two primary doses, one each at ages 6 weeks and 14 weeks (2 + 1 groups). All infants received a booster dose at age 40 weeks. PCV=pneumococcal conjugate vaccine. PCV10=ten-valent PCV. PCV13=13-valent PCV. NA=not applicable.

* Data not available for all randomly assigned infants.

**Figure 2:**
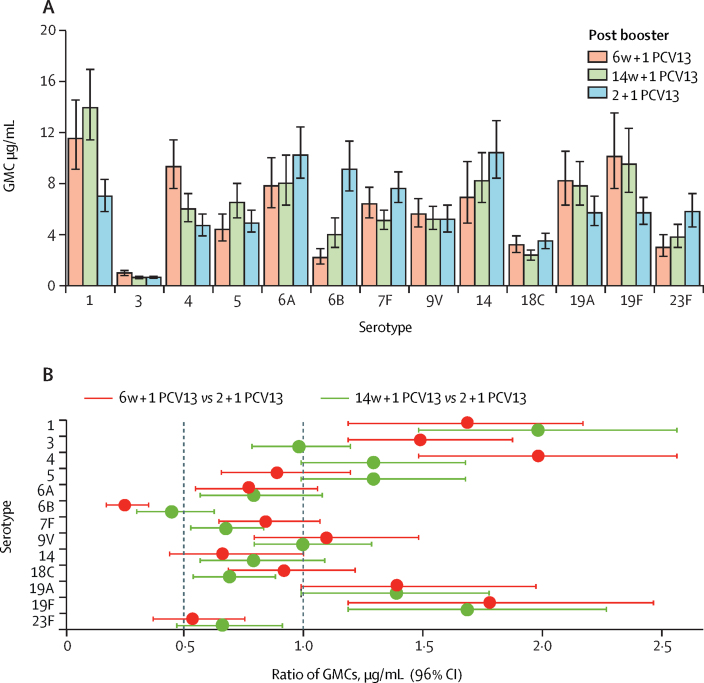
Serum IgG 1 month post booster with PCV13 following a single-dose or two-dose primary series (A) GMCs of serotype-specific IgG antibodies (error bars indicate 96% CIs). (B) Ratio of serotype-specific GMCs. The vertical dashed line at 0·5 indicates the non-inferiority margin; for the 1 + 1 vaccine schedule to be non-inferior to the 2 + 1 schedule, the lower bound of the 96% CI for the ratio of GMCs had to be higher than 0·5 for at least ten of the 13 vaccine serotypes. The serotype-specific IgG GMC was higher in the 1 + 1 group than in the 2 + 1 group if the lower bound of the 96% CI was above 1, whereas the serotype-specific IgG GMC was lower in the 1 + 1 group than in the 2 + 1 group if the upper bound of the 96% CI was less than 1 (note that the limits have been rounded in this figure). Infants received one primary dose of PCV13 at age 6 weeks (6w + 1 PCV13) or 14 weeks (14w + 1 PCV13) or two primary doses, one each at ages 6 weeks and 14 weeks (2 + 1 PCV13). All infants received a booster dose of PCV13 at age 40 weeks. PCV13=13-valent pneumococcal conjugate vaccine. GMC=geometric mean concentration.

542 infants had serum samples available 1 month after the booster dose and were included in the modified intention-to-treat analysis of the primary endpoint. In PCV13-vaccinated infants, both 1 + 1 dosing schedules were non-inferior to the 2 + 1 schedule 1 month after booster vaccination (figure 2). The lower limit of the 96% CI for the ratio of IgG GMCs between the 1 + 1 and 2 + 1 groups was higher than 0·5 for ten serotypes (all except 6B, 14, and 23F) in the 6w + 1 PCV13 group and 11 serotypes (all except 6B and 23F) in the 14w + 1 PCV13 group. Post-booster IgG GMCs were higher in the 6w + 1 PCV13 group than in the 2 + 1 PCV13 group for serotypes 1, 3, 4, 19A, and 19F, and lower in the 6w+1 PCV13 group than in the 2 + 1 PCV13 group for serotypes 6B and 23F (figure 2). The 14w + 1 PCV13 group had higher IgG GMCs than the 2 + 1 PCV13 group for serotypes 1, 19A, and 19F, and lower IgG GMCs for serotypes 6B, 7F, 18C, and 23F (figure 2; appendix pp 4–5).

**Figure 3:**
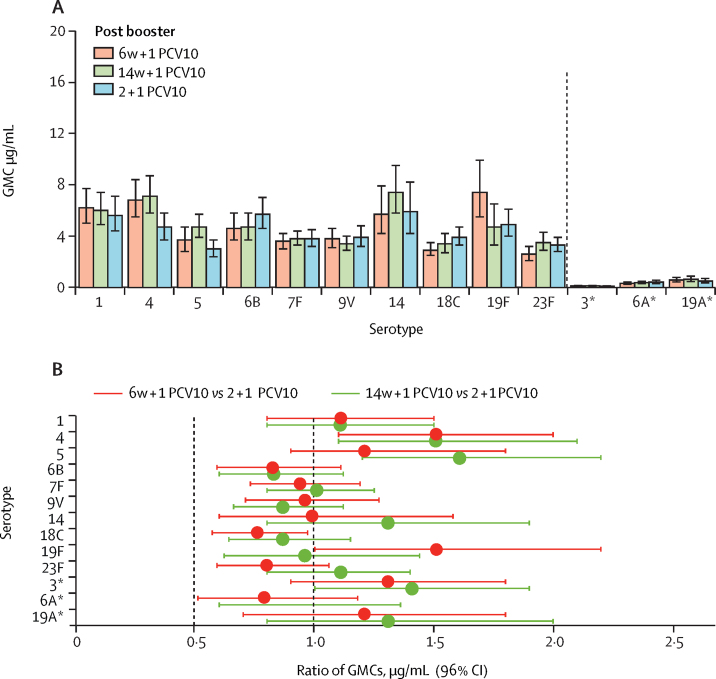
Serum IgG 1 month post booster with PCV10 following a single-dose or two-dose primary series (A) GMCs of serotype-specific IgG antibodies (error bars indicate 96% CIs). (B) Ratio of serotype-specific GMCs. The vertical dashed line at 0·5 indicates the non-inferiority margin; for the 1 + 1 vaccine schedule to be non-inferior to the 2 + 1 schedule, the lower bound of the 96% CI for the ratio of GMCs had to be higher than 0·5 for at least eight of the ten vaccine serotypes. The serotype-specific IgG GMC was higher in the 1 + 1 group than in the 2 + 1 group if the lower bound of the 96% CI was above 1, whereas the serotype-specific IgG GMC was lower in the 1 + 1 group than in the 2 + 1 group if the upper bound of the 96% CI was less than 1 (note that the limits have been rounded in this figure). Infants received one primary dose of PCV10 at age 6 weeks (6w + 1 PCV10) or 14 weeks (14w + 1 PCV10) or two primary doses, one each at ages 6 weeks and 14 weeks (2 + 1 PCV10). All infants received a booster dose of PCV10 at age 40 weeks. PCV10=ten-valent pneumococcal conjugate vaccine. GMC=geometric mean concentration. *Serotypes included in the 13-valent but not the ten-valent pneumococcal conjugate vaccine.

In PCV10-vaccinated infants, both 1 + 1 dosing schedules were non-inferior to the 2 + 1 dosing schedule; the lower limit of the 96% CI for the ratio of GMCs between the 1 + 1 and 2 + 1 groups was higher than 0·5 for all ten PCV10 serotypes in both 1 + 1 groups (figure 3). Higher IgG GMCs 1 month after the booster dose were observed with a 1 + 1 schedule than with a 2 + 1 schedule of PCV10 for serotypes 4 (both 1 + 1 PCV10 groups), 5 (14w + 1 PCV10 group only), and 19F (6w + 1 PCV10 group only). Post-booster GMCs were lower for serotype 18C in the 6w + 1 PCV10 group than in the 2 + 1 PCV10 group but similar for the remaining serotypes (figure 3; appendix pp 6–7).

Comparing post-booster immune responses between the 1 + 1 PCV13 groups in a post-hoc analysis, GMCs were lower for serotypes 3, 4, and 18C, and higher for serotypes 5 and 6B, in the 14w + 1 group than in the 6w + 1 group (appendix pp 4–5). In PCV10-vaccinated groups, post-booster GMCs were similar for nine of the ten PCV10 serotypes between the 6w + 1 and 14w + 1 groups; GMCs were lower for serotype 19F in the 14w + 1 group than in the 6w + 1 group (appendix pp 6–7).

Proportions of PCV13-vaccinated infants with serotype-specific IgG concentrations of at least 0·35 μg/mL 1 month after the booster dose were at least 94·4% for all serotypes in all groups except for serotype 3 (range 79·8–85·9%) in all groups and serotype 6B (88·0%) in the 6w + 1 group (appendix pp 4–5). Non-inferiority was shown for both PCV13 1 + 1 groups compared with the 2 + 1 group when evaluating seroprotection. The lower limit of the 96% CI for the difference in percentage was at least −10% for ten serotypes in the 6w + 1 PCV13 group (all except serotypes 3, 6B, and 14) and 11 serotypes in the 14w + 1 PCV13 group (all except serotypes 3 and 6B; appendix pp 4–5).

For PCV10-vaccinated infants, at least 90·3% had post-booster serotype-specific IgG concentrations of at least 0·35 μg/mL for all ten serotypes across all three groups, except for 19F (88·4%) in the 14w + 1 group (appendix pp 6–7). The lower limit of the 96% CI for the difference in proportions was at least −10% for nine serotypes in the 6w + 1 PCV10 group (all except serotype 14) and eight in the 14w + 1 PCV10 group (all except serotypes 14 and 19F). The percentage of infants with serotype 19F-specific IgG concentrations of at least 0·35 μg/mL was significantly lower in the 14w + 1 PCV10 group than in the 2 + 1 PCV10 group (88·4% *vs* 98·9%; p=0·0042; appendix pp 6–7).

Post-booster OPA geometric mean titres (GMTs) were generally similar (overlap of 95% CI) between the 1 + 1 and and 2 + 1 groups for most serotypes and for both vaccines (appendix pp 7–8). The majority of PCV13-vaccinated infants (90–100%) and PCV10-vaccinated infants (89·5–100%) had detectable OPA activity (ie, greater or equal to the lower limit of assay quantification, appendix pp 8–9).

In PCV13-vaccinated infants, IgG GMCs were lower for all serotypes (except for serotype 3) in the 1 + 1 groups than in the 2 + 1 group 1 month after completion of the primary series (appendix pp 10–11). The percentage of infants with serotype-specific IgG concentrations of at least 0·35 μg/mL in the 2 + 1 PCV13 group ranged between 61·1% (serotype 6B) and 96·8% (serotypes 7F and 19F). Both 1 + 1 PCV13 groups were inferior to the 2 + 1 group when comparing the proportions of infants with serotype-specific IgG concentrations of at least 0·35 μg/mL (appendix pp 10–11). Proportions were significantly lower in the 1 + 1 groups than in the 2 + 1 group for all serotypes, except for serotypes 3 and 19F in both 1 + 1 groups and serotype 14 in the 14w + 1 group.

**Figure 4:**
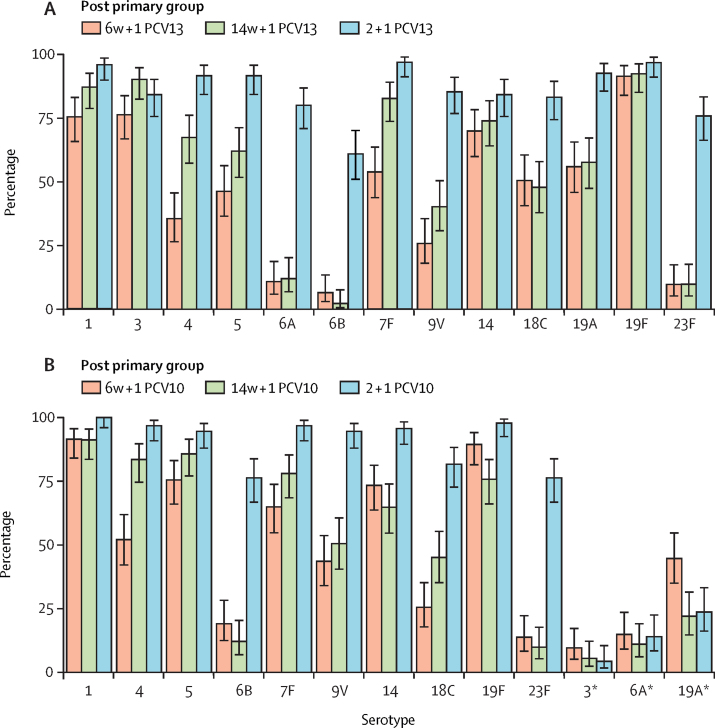
Percentage of infants with serotype-specific serum IgG concentrations ≥0·35 μg/mL 1 month after a single-dose or two-dose primary series with PCV13 (A) or PCV10 (B) Error bars show 96% CI (note that the limits have been rounded in this figure). Infants received one primary dose of PCV10 or PCV13 at age 6 weeks (6w + 1 PCV10 or PCV13) or 14 weeks (14w + 1 PCV10 or PCV13) or two primary doses, one each at ages 6 weeks and 14 weeks (2 + 1 groups). PCV13=13-valent pneumococcal conjugate vaccine. PCV10=ten-valent pneumococcal conjugate vaccine. *Serotypes included in PCV13 but not in PCV10.

Post-primary dose IgG GMCs were higher in the 14w + 1 PCV13 group than in the 6w + 1 PCV13 group for serotypes 1, 3, 4, 5, 7F, and 9V based on the 96% CIs, and infants in the 14w + 1 PCV13 group were also more likely than those in the 6w+1 group to have IgG concentrations of at least 0·35 μg/mL for these serotypes (excluding serotype 1; figure 4A; appendix pp 10–11).

Among PCV10-vaccinated infants, post-primary series IgG GMCs were higher for all vaccine serotypes in the 2 + 1 group than in the 1 + 1 groups (appendix pp 12–13). The proportion of infants with serotype-specific IgG concentrations of at least 0·35 μg/mL at this timepoint was significantly higher for all ten vaccine serotypes in the 2 + 1 PCV10 group than in either 1 + 1 PCV10 group (excluding serotype 5; appendix pp 12–13). There was no consistent pattern in differences in GMCs between the 6w + 1 PCV10 and 14w + 1 PCV10 groups, with GMCs lower in the 14w + 1 group than in the 6w + 1 group for serotypes 6B, 14, and 19F, and higher in the 14w + 1 group than in the 6w + 1 group for serotypes 4, 7F, and 18C (appendix pp 12–13). Furthermore, a similar proportion of infants in the 6w + 1 and 14w + 1 groups had IgG concentrations of at least 0·35 μg/mL for most serotypes; exceptions were lower percentages for serotypes 4 and 18C and a higher percentage for 19F in the 6w + 1 than in the 14w + 1 groups (figure 4B; appendix pp 12–13).

**Figure 5:**
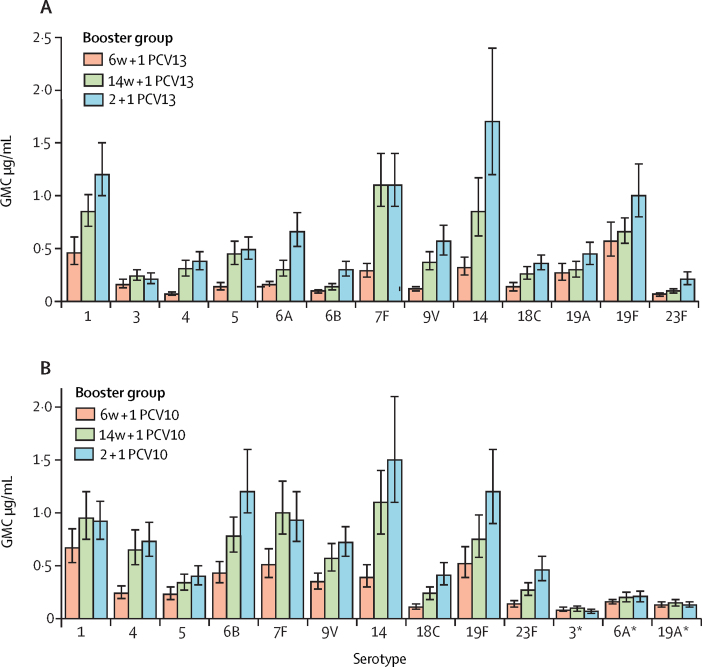
Serum IgG GMCs pre-booster dose with PCV13 (A) or PCV10 (B) following a single-dose or two-dose primary series Error bars show 96% CIs (note that the limits have been rounded in this figure). Infants received one primary dose of PCV10 or PCV13 at age 6 weeks (6w + 1 PCV10 or PCV13) or 14 weeks (14w + 1 PCV10 or PCV13) or two primary doses, one each at ages 6 weeks and 14 weeks (2 + 1 groups), plus booster doses at age 40 weeks. PCV13=13-valent pneumococcal conjugate vaccine. PCV10=ten-valent pneumococcal conjugate vaccine. GMC=geometric mean concentration. *Serotypes included in PCV13 but not in PCV10.

In PCV13-vaccinated infants, the pre-booster GMCs and percentages of infants with serotype-specific IgG concentrations of at least 0·35 μg/mL were lower for all serotypes (excluding serotype 3) in the 6w + 1 group than in the 2 + 1 group (figure 5A; appendix pp 14–15). By contrast, the lower limit of the 96% CI for the ratio of GMCs between the 14w + 1 and 2 + 1 groups was higher than 0·5 for six serotypes (1, 3, 4, 5, 7F, and 18C), and the proportion of infants with IgG concentrations of at least 0·35 μg/mL was similar between the groups for ten serotypes (all except 6A, 6B, and 23F; figure 5A; appendix pp 14–15).

Infants in the 14w + 1 PCV13 group had higher pre-boost GMCs than did infants in the 6w + 1 PCV13 group for 11 serotypes (all except for 19A and 19F) and were more likely than infants in the 6w + 1 PCV13 group to have IgG concentrations of at least 0·35 μg/mL for most PCV13 serotypes (all except for 6B, 19A, and 23F; figure 5A; appendix pp 14–15).

In infants vaccinated with PCV10, pre-booster GMCs and percentages of infants with IgG concentrations of at least 0·35 μg/mL were lower for all vaccine serotypes in the 6w + 1 group than in the 2 + 1 group (except for serotype 1; figure 5B; appendix pp 16–17). In the 14w + 1 group, pre-booster GMCs were similar to those in the 2 + 1 group for six PCV10 serotypes (1, 4, 5, 7F, 9V, and 14) and lower than those in the 2 + 1 group for four PCV10 serotypes (6B, 18C, 19F, and 23F). A similar proportion of infants in the 14w + 1 group as in the 2 + 1 group had pre-booster IgG concentrations of at least 0·35 μg/mL for eight (1, 4, 5, 6B, 7F, 9V, 14, and 19F) of the PCV10 serotypes. Furthermore, the 14w + 1 PCV10 group had higher pre-booster GMCs than the 6w + 1 PCV10 group for eight serotypes (all except serotypes 1 and 19F) and was more likely than the 6w + 1 group to have IgG concentrations of at least 0·35 μg/mL for these serotypes (except serotype 5; figure 5B; appendix pp 16–17).

During the study period, 84 serious adverse events were reported in 72 (12%) of 600 participants. Of these, 15 occurred within 28 days of vaccination, but none were considered to be related to PCV injection. There were no cases of culture-confirmed invasive pneumococcal disease, and the rates of all-cause lower respiratory tract infections (bronchiolitis, pneumonia, and viral pneumonia) were similar between the groups (appendix p 18).

## Discussion

The primary outcome of this study was post-booster dose immunogenicity of a 1 + 1 compared with a 2 + 1 PCV dosing schedule rather than immune responses after the primary series. The rationale for the primary outcome was premised on the inverse association between serotype-specific antibody concentrations and colonisation,^7^ and the fact that infants aged 1–4 years are the dominant source of pneumococcal transmission in the community.^8^, ^9^, ^10^, ^11^ In countries with an established PCV immunisation programme and where there has been substantial reduction or near elimination of vaccine-serotype colonisation across all age groups, it has been hypothesised that the PCV dosing schedule could be tailored and primarily aimed at sustaining the low prevalence of vaccine-serotype colonisation (and, by proxy, risk of invasive pneumococcal disease). This tailoring could include transitioning to a 1 + 1 dosing schedule as done in the UK in 2020.^15^

For PCV13, we found that post-booster GMCs of serotype-specific antibodies were non-inferior in both 1 + 1 groups compared with the 2 + 1 group. Moreover, post-booster OPA GMTs and percentages of infants with detectable OPA activity were similar between the 1 + 1 PCV13 and 2 + 1 PCV13 groups in a planned exploratory analysis. These findings corroborate those from the UK, which also reported equivalent or higher post-booster dose (at 12 months of age) GMCs against nine of the PCV13 serotypes in the 1 + 1 group (vaccination at 3 and 12 months of age) compared with the 2 + 1 group (vaccination at 2, 4, and 12 months of age).^21^

The UK study observed higher GMCs of antibodies to serotypes 1, 4, 14, and 19F in the 1 + 1 than in the 2 + 1 PCV13 groups.^21^ Similarly, we observed higher post-booster GMCs in both 1 + 1 PCV13 groups than in the 2 + 1 PCV13 group for serotypes 1, 19A, and 19F (as well as serotypes 3 and 4 in the 6w + 1 group). By contrast, post-booster IgG GMCs were lower for serotypes 6B and 23F in the 6w + 1 PCV13 group than in the 2 + 1 PCV13 group and for serotypes 6B, 7F, 18C, and 23F in the 14w + 1 PCV13 group. Lower post-booster GMCs were similarly observed in the PCV13 1 + 1 group than in the 2 + 1 groups for serotypes 6B and 23F in the UK.^21^ However, the similar OPA GMTs and proportions of infants with OPA titres above the lower limit of assay quantification between the 2 + 1 PCV13 and 1 + 1 PCV13 groups for serotypes 6B, 14, and 23F suggest that the differences in GMCs are unlikely to translate into increased risk for invasive pneumococcal disease. Ongoing surveillance would be needed to ascertain whether serotype-specific differences in GMCs (higher or lower) between the 1 + 1 PCV13 and 2 + 1 PCV13 groups affect vaccine-serotype colonisation and incidence of invasive pneumococcal disease.

We also observed non-inferiority in the post-booster GMCs for the 6w + 1 PCV10 and 14 + 1 PCV10 groups compared with the 2 + 1 PCV10 group. Generally, the post-booster GMCs were similar for the 1 + 1 PCV10 groups and the 2 + 1-PCV10 group.

We investigated whether delaying the first PCV dose in a 1 + 1 dosing schedule from 6 to 14 weeks of age would enhance immunogenicity following the priming dose premised on the hypotheses that transplacentally acquired serotype-specific IgG might interfere with immune responses to vaccines to a greater extent the closer to time of birth,^22^ maturation of the infant's immune system during the first few weeks of life possibly enhances immune responses to vaccines, and previous vaccination with a diphtheria toxoid-containing vaccine (hexaxim at 6 and 10 weeks of age in this study) might heighten the immune response to PCV conjugated to carrier-related molecule 197 (a diphtheria toxoid-like molecule). GMCs of serotype-specific antibodies 1 month after the single primary dose of PCV13 were higher in the 14w + 1 group than in the 6w + 1 group for six serotypes, and for 1 + 1 serotypes the proportion of infants with IgG concentrations of at least 0·35 μg/mL was higher in the 14w + 1 group than in the 6w + 1 group. These differences persisted through to 9 months of age (pre-booster assessment). Furthermore, pre-booster GMCs of serotype-specific antibodies were similar in the 14w + 1 and 2 + 1 groups for four serotypes and the proportions of infants with IgG concentrations of at least 0·35 μg/mL were similar in the 14w + 1 and 2 + 1 groups for ten serotypes. By contrast, both of these measures were lower for 12 serotypes (except serotype 3) in the 6w + 1 PCV13 group than in the 2 + 1 PCV13 group.

There were no consistent differences between the 6w + 1 PCV10 group and the 14w + 1 PCV10 group in GMCs of serotype-specific antibodies or percentages of infants with IgG concentrations of 0·35 μg/mL or higher 1 month after the single primary dose. For PCV10 serotype 18C (the only serotype conjugated to diphtheria toxoid), the post-first dose GMCs (and percentages of infants with IgG ≥0·35 μg/mL) were, however, higher in the 14w + 1 group than in the 6w + 1 group; by contrast, they were lower in the 14w + 1 group than in the 6w+1 group for serotype 19F (conjugated to tetanus toxoid). This finding suggests that the heightened immune response in the 14w + 1 PCV13 group compared with the 6w + 1 PCV13 group was probably due to priming by antecedent immunisation with a diphtheria-containing vaccine.^23^

The 14w + 1 PCV10 group had higher IgG GMCs (for nine PCV10 serotypes) and was more likely to have IgG concentrations of at least 0·35 μg/mL (for eight serotypes) than the 6w + 1 PCV10 group at 9 months of age (pre-booster assessment). Additionally, compared with the 2 + 1 PCV10 group, pre-booster GMCs and proportions of infants with IgG concentrations of at least 0·35 μg/mL were similar in the 14w + 1 PCV10 group for six and eight serotypes, respectively, and uniformly lower for all PCV10 serotypes in the 6w + 1 PCV10 group. The narrower window between vaccination and the pre-booster dose timepoint in the 14w + 1 group (approximately 26 weeks) than in the 6w + 1 group (approximately 34 weeks) might have contributed in part to the higher GMCs of serotype-specific antibodies pre-booster dose in the 14w + 1 group than in the 6w + 1 group.

These findings indicate that delaying the first PCV13 or PCV10 dose when adopting a 1 + 1 dosing schedule could offset some of the reduced immunity compared with a two-dose primary series, at least up to the time of the booster dose. Also, the possible immunity gap resulting from delaying PCV immunisation by 8 weeks (ie, from 6 to 14 weeks of age) might be mitigated by the 1 + 1 dosing schedule possibly being as effective as a 2 + 1 PCV schedule in sustaining the already low prevalence of vaccine-serotype colonisation and transmission in the community.^8^, ^14^ This transition from a 2+1 to a 1+1 dosing regimen would probably continue to confer indirect protection against pneumococcal disease in these young PCV-unvaccinated infants.^12^, ^24^ In South Africa, before routine childhood PCV immunisation was introduced, 14% of overall invasive pneumococcal disease in infants occurred in the first 10 weeks of life (54% being PCV10 serotypes and 74% being PCV13 serotypes).^24^ Within 4 years of PCV7/13 (PCV7 was introduced in 2009 and became PCV13 in 2010) introduction into the routine infant immunisation programme, the incidence of PCV13-serotype invasive pneumococcal disease declined by 78% (95% CI 60–88) in South African infants under 10 weeks of age.^13^ In addition, although evidence on effectiveness of a single dose of PCV is conflicting, the effectiveness of a single PCV dose in early infancy could be as high as 73%.^3^, ^25^, ^26^ Furthermore, the theoretical risk of increased susceptibility to invasive pneumococcal disease in the 14w + 1 PCV13 group compared with the 6w + 1 PCV13 group for the intervening period when vaccination is delayed might be offset by the subsequent heightened protection against invasive pneumococcal disease in the 14w + 1 PCV13 group during the subsequent 26 weeks leading up to the PCV booster dose at 40 weeks of age.

Our study provides supporting immunological evidence for the case of transitioning from a 2 + 1 to a 1 + 1 PCV13 or PCV10 dosing schedule, including when the booster dose is given as early as 40 weeks of age. However, we did not power the study to evaluate the relative efficacy of a 1 + 1 compared with 2 + 1 PCV dosing schedule against either pneumococcal disease or colonisation. The safety data, however, did not suggest any difference between the groups in illnesses such as all-cause pneumonia, which could be due to pneumococcal infection. Additional data on the epidemiology of pneumococcal colonisation and disease are required from our setting (and other similar low-income and middle-income countries) in which the 2 + 1 PCV childhood immunisation programme has existed since 2009 to inform deliberations on whether transitioning to a 1 + 1 schedule should be considered, as the dynamics of and effect of PCV on pneumococcal colonisation could differ by income setting.^9^ Although pneumococcal colonisation occurs at a much earlier age in low-income and middle-income countries than in high-income countries, children aged 1–4 years are still considered to be the dominant transmitters of pneumococci.^11^ In South Africa, vaccine-serotype colonisation decreased by approximately two-thirds in children younger than 5 years and approximately 55% in adults living with and without HIV within 3–4 years of introducing the 2 + 1 PCV7/13 dosing schedule into the public immunisation programme.^27^, ^28^, ^29^ Additional follow-up colonisation studies are under way in urban and rural low-income South African settings. In addition, there is ongoing nationwide surveillance for invasive pneumococcal disease across all age groups.^13^, ^30^ These additional sources of information, coupled with a cost analysis and benefit–risk assessment and infrastructure to deliver high PCV immunisation coverage for the primary and booster doses of a 1 + 1 schedule, are required to ascertain whether South Africa (and other countries of similar settings) should consider transitioning to a 1 + 1 PCV dosing schedule as has been done in the UK.

Factors contributing to the UK transitioning from a 2 + 1 to a 1 + 1 PCV13 dosing schedule included near elimination of PCV13-serotype (except serotype 3) invasive pneumococcal disease in vaccinated and unvaccinated age groups since the introduction of childhood PCV immunisation in 2006, low residual prevalence of PCV13-serotype colonisation in the population, non-inferior post-booster immunogenicity in children vaccinated with a 1 + 1 compared with a 2 + 1 PCV13 dosing series, and the opportunity to reduce the number of injectable vaccines in the infant immunisation programme.^8^, ^15^, ^21^ A mathematical modelling study in the UK concluded that transitioning from the 2 + 1 to the 1 + 1 PCV13 dosing schedule would result in an average of 31 additional cases of vaccine-serotype invasive pneumococcal disease across all age groups over a 5-year period and no increase in pneumococcal deaths in children younger than 15 years.^8^

Decreasing the number of PCV doses would reduce the cost of PCV procurement, which has remained largely unchanged since the vaccine was first licensed in 2000, and is among the most expensive vaccines procured in the South African (and probably other middle-income settings) public immunisation programme. The high cost of PCV has probably contributed to many middle-income countries not having introduced PCV into their public immunisation programmes.^31^ Addressing this barrier, either through price reduction or decreasing the number of PCV doses (once having established a successful PCV programme by means of a 2 + 1 or three-dose primary series dosing schedule), is also relevant to the approximately 32 countries (including 11 African countries) that will need to transition out of Gavi, the Vaccine Alliance, financial support for PCV procurement when they exceed the gross domestic product income threshold that Gavi uses to determine elligibility for support; these countries will need to establish funding models for the continued use of PCV in their public immunisation programmes.^32^

**This online publication has been corrected. The corrected version first appeared at thelancet.com/infection on September 11, 2020**

## Data sharing

In keeping with the Gates Foundation policy on open data access, data will be shared on request from the corresponding author on a collaborative basis. Individual participant data used in this manuscript will be provided, after removal of personal identifiers, as well as the necessary data dictionary.

## References

[1] WHO (2012). Pneumococcal vaccines WHO position paper—2012. Wkly Epidemiol Rec.

[2] Conklin L, Loo JD, Kirk J (2014). Systematic review of the effect of pneumococcal conjugate vaccine dosing schedules on vaccine-type invasive pneumococcal disease among young children. Pediatr Infect Dis J.

[3] Whitney CG, Pilishvili T, Farley MM (2006). Effectiveness of seven-valent pneumococcal conjugate vaccine against invasive pneumococcal disease: a matched case-control study. Lancet.

[4] Jayasinghe S, Chiu C, Quinn H, Menzies R, Gilmour R, McIntyre P (2018). Effectiveness of 7- and 13-valent pneumococcal conjugate vaccines in a schedule without a booster dose: a 10-year observational study. Clin Infect Dis.

[5] Klugman KP, Madhi SA, Adegbola RA, Cutts F, Greenwood B, Hausdorff WP (2011). Timing of serotype 1 pneumococcal disease suggests the need for evaluation of a booster dose. Vaccine.

[6] Käyhty H, Auranen K, Nohynek H, Dagan R, Mäkelä H (2006). Nasopharyngeal colonization: a target for pneumococcal vaccination. Expert Rev Vaccines.

[7] Voysey M, Fanshawe TR, Kelly DF (2018). Serotype-specific correlates of protection for pneumococcal carriage: an analysis of immunity in 19 countries. Clin Infect Dis.

[8] Choi YH, Andrews N, Miller E (2019). Estimated impact of revising the 13-valent pneumococcal conjugate vaccine schedule from 2 + 1 to 1 + 1 in England and Wales: a modelling study. PLoS Med.

[9] Choi YH, Melegaro, A, van Hoek AJ, Roca A, Mackenzie G, Gay N. Impact of thirteen-valent pneumococcal conjugate vaccine on pneumococcal carriage in different countries—mathematical modelling study. Poster presentation at ISPPD-9; Hyderabad, India, 2014.

[10] Le Polain de Waroux O, Flasche S, Prieto-Merino D, Edmunds WJ (2014). Age-dependent prevalence of nasopharyngeal carriage of *Streptococcus pneumoniae* before conjugate vaccine introduction: a prediction model based on a meta-analysis. PLoS One.

[11] Shiri T, Auranen K, Nunes MC (2013). Dynamics of pneumococcal transmission in vaccine-naive children and their HIV-infected or HIV-uninfected mothers during the first 2 years of life. Am J Epidemiol.

[12] Shiri T, Datta S, Madan J (2017). Indirect effects of childhood pneumococcal conjugate vaccination on invasive pneumococcal disease: a systematic review and meta-analysis. Lancet Glob Health.

[13] von Gottberg A, de Gouveia L, Tempia S (2014). Effects of vaccination on invasive pneumococcal disease in South Africa. N Engl J Med.

[14] Flasche S, Van Hoek AJ, Goldblatt D (2015). The potential for reducing the number of pneumococcal conjugate vaccine doses while sustaining herd immunity in high-income countries. PLoS Med.

[15] Joint Committee on Vaccination and Immunisation Minutes of the Joint Committee on Vaccination and Immunisation meeting on Oct 4, 2017. https://www.gov.uk/government/groups/joint-committee-on-vaccination-and-immunisation#minutes.

[16] Wernette CM, Frasch CE, Madore D (2003). Enzyme-linked immunosorbent assay for quantitation of human antibodies to pneumococcal polysaccharides. Clin Diagn Lab Immunol.

[17] Goldblatt D, Plikaytis BD, Akkoyunlu M (2011). Establishment of a new human pneumococcal standard reference serum, 007sp. Clin Vaccine Immunol.

[18] Rose CE, Romero-Steiner S, Burton RL (2011). Multilaboratory comparison of *Streptococcus pneumoniae* opsonophagocytic killing assays and their level of agreement for the determination of functional antibody activity in human reference sera. Clin Vaccine Immunol.

[19] Siber GR, Chang I, Baker S (2007). Estimating the protective concentration of anti-pneumococcal capsular polysaccharide antibodies. Vaccine.

[20] Rüger B (1978). Das maximale signifikanzniveau des Tests: Lehne*H*_0_ ab, wenn *k* unter *n* gegebenen tests zur ablehnung führen. Metrika.

[21] Goldblatt D, Southern J, Andrews NJ (2018). Pneumococcal conjugate vaccine 13 delivered as one primary and one booster dose (1 + 1) compared with two primary doses and a booster (2 + 1) in UK infants: a multicentre, parallel group randomised controlled trial. Lancet Infect Dis.

[22] Scott JA, Ojal J, Ashton L, Muhoro A, Burbidge P, Goldblatt D (2011). Pneumococcal conjugate vaccine given shortly after birth stimulates effective antibody concentrations and primes immunological memory for sustained infant protection. Clin Infect Dis.

[23] Tashani M, Jayasinghe S, Harboe ZB, Rashid H, Booy R (2016). Potential carrier priming effect in Australian infants after 7-valent pneumococcal conjugate vaccine introduction. World J Clin Pediatr.

[24] von Gottberg A, Cohen C, de Gouveia L (2013). Epidemiology of invasive pneumococcal disease in the pre-conjugate vaccine era: South Africa, 2003–2008. Vaccine.

[25] Gidding HF, McCallum L, Fathima P (2018). Effectiveness of a 3 + 0 pneumococcal conjugate vaccine schedule against invasive pneumococcal disease among a birth cohort of 1·4 million children in Australia. Vaccine.

[26] Cohen C, von Mollendorf C, de Gouveia L (2017). Effectiveness of the 13-valent pneumococcal conjugate vaccine against invasive pneumococcal disease in South African children: a case-control study. Lancet Glob Health.

[27] Nzenze SA, Madhi SA, Shiri T (2017). Imputing the direct and indirect effectiveness of childhood pneumococcal conjugate vaccine against invasive pneumococcal disease by surveying temporal changes in nasopharyngeal pneumococcal colonization. Am J Epidemiol.

[28] Nzenze SA, von Gottberg A, Shiri T (2015). Temporal changes in pneumococcal colonization in HIV-infected and HIV-uninfected mother–child pairs following transitioning from 7-valent to 13-valent pneumococcal conjugate vaccine, Soweto, South Africa. J Infect Dis.

[29] Nzenze SA, Shiri T, Nunes MC (2013). Temporal changes in pneumococcal colonization in a rural African community with high HIV prevalence following routine infant pneumococcal immunization. Pediatr Infect Dis J.

[30] National Institute for Communicable Diseases Cumulative invasive pneumococcal disease case numbers reported by the GERMS-SA surveillance programme, 1 January 2012 to 30 April 2019. http://www.nicd.ac.za/wp-content/uploads/2019/05/IPD_cumulative_9May2019_v2-002.pdf.

[31] Tricarico S, McNeil HC, Cleary DW (2017). Pneumococcal conjugate vaccine implementation in middle-income countries. Pneumonia (Nathan).

[32] Fridh Å, Bastin J, Bertot E Gavi: 2017 progress report. https://www.gavi.org/sites/default/files/publications/progress-reports/Gavi-Progress-Report-2017.pdf.

