# Correction to *Lancet Infect Dis* 2020; published online Aug 25. https://doi.org/10.1016/S1473-3099(20)30289-9

**DOI:** 10.1016/S1473-3099(20)30741-6

**Published:** 2020-11

*Madhi SA, Mutsaerts EAML, Izu A, et al. Immunogenicity of a single-dose compared with a two-dose primary series followed by a booster dose of ten-valent or 13-valent pneumococcal conjugate vaccine in South African children: an open-label, randomised, non-inferiority trial.* Lancet Infect Dis *2020; published online Aug 25. https://doi.org/10.1016/S1473-3099(20)30289-9*—In figure 4a of this Article, a bar related to serotype 6B in the 2 + 1 PVC13 group was omitted. This correction has been made to the online version as of Sept 11, 2020 and will be made to the printed version.

