# Supplementary material for: Immunogenicity of a single-dose compared with a two-dose primary series followed by a booster dose of ten-valent or 13-valent pneumococcal conjugate vaccine in South African children: an open-label, randomised, non-inferiority trial
Source: Lancet Infect Dis. 2020 Dec;20(12):1426–36. doi: 10.1016/S1473-3099(20)30289-9 (PMC7689288; doi:10.1016/S1473-3099(20)30289-9)
Supplement: Supplementary appendix [file mmc1.pdf]

THE LANCET Infectious Diseases

Supplementary webappendix

This webappendix formed part of the original submission and has been peer reviewed. We post it as supplied by the authors.

Supplement to: Madhi SA, Mutsaert EAML, Izu A, et al. Immunogenicity of a single-dose compared with a two-dose primary series followed by a booster dose of ten-valent or 13-valent pneumococcal conjugate vaccine in South African children: an open-label, randomised, non-inferiority trial. *Lancet Infect Dis* 2020; published online Aug 25. https://doi.org/10.1016/S1473-3099(20)30289-9.

Supplementary text: Immunogenicity of a single compared to two-dose primary series followed by a booster dose of 10-valent or 13-valent pneumococcal conjugate vaccine in South African children: An open-label, randomised, non-inferiority trial.

Shabir A Madhi,[1,2] Eleonora AML Mutsaerts*,[1,2] Alane Izu,[1,2] Welekazi Boyce,[1,2] Sutika Bhikha,[1,2] Benit T Ikulinda,[1,2] Lisa Jose,[1,2] Anthonet Koen,[1,2] Amit J. Nana,[1,2] Andrew Moultrie,[1,2] Lucy Roalfe,[3] Adam Hunt,[3] David Goldblatt,[3] Clare L Cutland, [1,2], Jeffrey R Dorfman*[1,2]

[1] Medical Research Council: Respiratory and Meningeal Pathogens Research Unit, University of the Witwatersrand, Faculty of Health Science, Johannesburg, South Africa

[2] Department of Science/ National Research Foundation: Vaccine Preventable Diseases, University of the Witwatersrand, Faculty of Health Science, Johannesburg, South Africa

[3] Immunobiology Section, University College of London, Great Ormond Street Institute of Child Health Biomedical Research Centre, London, UK

Correspondence to: Professor Shabir A Madhi. RMPRU, 11th Floor, Central-West Wing, Chris Hani Baragwanath Academic Hospital, Chris Hani Road, Soweto, 2013, South Africa.

E-mail: madhis@rmpru.co.za

Tel: +27-11-9834266

1.1. Inclusion and Exclusion criteria:

Inclusion criteria included birth at ≥37 weeks gestation, birth weight ≥2500 grams and weighing ≥3500 grams at time of enrolment. Exclusion criteria included any clinically significant congenital abnormalities, any previous hospitalisation for respiratory illness of history confirmed pneumococcal disease since birth. Other exclusion criteria included receipt of any other investigational drug/vaccine, any previous history of PCV vaccination, known allergy to any of the vaccine components or febrile illness at time of enrolment, planned relocation outside of the study area up to age of two years, receipt of blood transfusion or any other blood products since birth, and any known or suspected immunodeficiency condition.

1.2. Vaccination of study participants

Children received either two or three intramuscular injections (0.5 mL) of PCV10 (Synflorix®, GlaxoSmithKline, Rixensart, Belgium) or PCV13 (Prevnar-13®, Pfizer, New York City, United States) in the anterolateral thigh at the vaccination visits. In addition, children received routine vaccinations according to the South African public immunisation schedule: combined diphtheria, tetanus, pertussis, hepatitis B, poliomyelitis and *Haemophilus influenzae* type b conjugate vaccine (Hexaxim®, Sanofi Pasteur, Paris, France) at 6, 10, 14 weeks and 15 months of age, oral rotavirus vaccine (Merieux®, Institut Merieux, Lyon, France) at 6 and 14 weeks of age, and measles vaccine at 6 and 12 months of age (Measbio®, The Biovac Institute, Cape Town, South Africa). Participants were observed for 30 minutes after administration of each vaccine by a study doctor or nurse for immediate reactions.

1.3. Serotype antibody ELISA assay method

All lots of reagents and 96 well plates were tested with the 12 WHO calibration sera and rejected if measured values did not conform to established values. Serotype-specific immunoglobulin G (IgG) concentrations were measured to all PCV13 capsular polysaccharides (1, 3, 4, 5, 6A, 6B, 7F, 9V, 14, 18C, 19A, 19F, and 23F) for all groups, PCV13 and PCV10 immunised. All serum samples were pre-adsorbed with cell wall polysaccharide, 22F polysaccharide and bovine serum albumin. Plates were coated with pre-tested amounts of serotype-specific carbohydrates (ATCC, Rockville, USA, and for serotype 1 also Statens Serum Institute, Copenhagen, Denmark), and were developed with alkaline phosphatase-conjugated secondary reagents (Life Technologies) that were pre-tested to confirm minimal reactivity to human IgM or IgA and equivlent reactivity to IgGsubclasses. Reagents and 96 well plates were tested with the 12 WHO calibration sera (ref 17) and rejected if measured values did not conform to established values. Limits of detection (LODs) were

calculated for each ELISA plate lot and were all below 0.20µg/mL for all serotypes except 6A (range 0.14-0.25µg/mL) and 14 (range 0.13-0.28µg/mL). LODs for serotypes 3, 5 and 18C were all below 0.10µg/mL.1,4. Additional secondary study objectives included:

i. Reporting on the proportion of study participants with vaccine-serotype specific serum IgG antibody concentration above the serotype-specific correlate for invasive pneumococcal disease (IPD) proposed by Andrews et al.[1] Furthermore, we planned on comparing the proportion in each group with post-booster dose vaccine-serotype specific serum IgG antibody concentration above arbitrarily selected thresholds of ≥0.5µg/ml and ≥1.0µg/ml as an exploratory objective. Due to the density and complexity of data presented in the manuscript, we have limited only reporting statistics in relation to the WHO proposed putative threshold for correlate of protection (≥0.35µg/ml) in this manuscript. Future publicatiions will report on te alternate thresholds indicated above.
ii. Furthermore, we also plan to report separately on the addional secondary objective comparing persistence of imunity meaaured at 18 months of age (i.e. 9 months post-booster; for which the antibody assay results are pending); to address the objective of analysing for the lower limit of the 95% confidence interval of the ratio of GMC of the 1+1 and 2+1 dosing schedules being >0.5 for individual serotypes at 18 months of age.
iii. The reporting for the secondary study objective comparing the PCV-1+1 to the PCV-2+1 dosing arms (stratified by PCV10 and PCV13) for prevalence of overall vaccine-serotype colonization at 9, 15 and 18 months of age will also be reported separately. The testing of the nasopharyngeal swab samples is currently underway using a novel multi-plex assay that has been undergoing expansion and validation at the site.[2]

Supplementary Table S1: Immune responses one-month following the booster dose of 13-valent pneumococcal conjugate vaccine (PCV13) in children who received a single dose at six (6w+1-PCV13) or 14 (14w+1-PCV13) weeks of age, and infants who received two doses at 6 and 14 weeks (2+1-PCV13) of age.

Measurement	Serotype	2+1-PCV13 n=92 [a]	6w+1-PCV13 n=92 [a]	Compare 6wk+1-PCV13 to 2+1-PCV13[b]	p-value[b]	14w+1-PCV13 n=89[a]	Compare 14wk+1-PCV13 to 2+1-PCV13[b]	p-value[b]	Comparing 14w+1-PCV13 to 6w+1-PCV13[c]	p-value[c]
GMC	1	7.0 (5.8-8.3)	11.5 (9.1-14.5)	1.66 (1.22-2.25)	0.0008	13.9 (11.4-16.9)	2 (1.52-2.63)	<0.0001	1.21 (0.88-1.66)	0.2229
	3	0.66 (0.58-0.75)	1.0 (0.8-1.2)	1.52 (1.19-1.94)	0.0005	0.65 (0.55-0.75)	0.98 (0.79-1.21)	0.8253	0.64 (0.5-0.83)	0.0005
	4	4.7 (3.9-5.6)	9.3 (7.6-11.4)	1.97 (1.48-2.61)	<0.0001	6 (5-7.2)	1.27 (0.98-1.65)	0.0590	0.65 (0.49-0.86)	0.0016
	5	4.9 (4.2-5.9)	4.4 (3.5-5.6)	0.89 (0.66-1.21)	0.4402	6.5 (5.3-8)	1.31 (0.99-1.74)	0.0485	1.47 (1.06-2.03)	0.0151
	6A	10.2 (8.4-12.4)	7.8 (6.1-10)	0.77 (0.55-1.07)	0.0980	8 (6.3-10.2)	0.79 (0.57-1.09)	0.1286	1.03 (0.72-1.47)	0.8815
	6B	9.1 (7.4-11.3)	2.2 (1.7-2.9)	0.24 (0.17-0.35)	<0.0001	4 (3-5.3)	0.44 (0.3-0.63)	<0.0001	1.81 (1.18-2.76)	0.0044
	7F	7.6 (6.5-8.9)	6.4 (5.3-7.7)	0.84 (0.65-1.08)	0.1468	5.1 (4.4-5.9)	0.67 (0.53-0.84)	0.0003	0.80 (0.62-1.03)	0.0702
	9V	5.2 (4.2-6.3)	5.6 (4.6-6.8)	1.09 (0.81-1.46)	0.5455	5.2 (4.4-6.2)	1.02 (0.77-1.34)	0.9067	0.93 (0.71-1.23)	0.6010
	14	10.4 (8.4-12.9)	6.9 (4.9-9.7)	0.66 (0.44-1.01)	0.0448	8.2 (6.5-10.4)	0.79 (0.57-1.1)	0.1450	1.19 (0.77-1.83)	0.4021
	18C	3.5 (2.9-4.1)	3.2 (2.6-3.9)	0.92 (0.69-1.23)	0.5536	2.4 (2-2.8)	0.69 (0.54-0.89)	0.0029	0.75 (0.57-0.99)	0.0333
	19A	5.7 (4.7-7)	8.2 (6.3-10.5)	1.42 (1.02-1.98)	0.0284	7.8 (6.3-9.7)	1.37 (1.01-1.85)	0.0329	0.96 (0.68-1.36)	0.8140
	19F	5.7 (4.8-6.9)	10.1 (7.6-13.5)	1.76 (1.24-2.52)	0.0012	9.5 (7.3-12.3)	1.66 (1.2-2.3)	0.0016	0.94 (0.63-1.41)	0.7539
	23F	5.8 (4.6-7.2)	3.0 (2.3-4.0)	0.53 (0.37-0.76)	0.0003	3.8 (3-4.8)	0.66 (0.47-0.92)	0.0102	1.24 (0.86-1.79)	0.2234
>=0.35	1	92; 100 (96.0-100)	91; 98.9 (94.1-99.8)	-1.09 (-4.81 - 2.69)	>0.9999	88; 98.9 (93.9-99.8)	-1.12 (-4.97 - 2.7)	0.4917	0.04 (-4.32 - 4.46)	>0.9999
	3	79; 85.9 (77.3-91.6)	79; 85.9 (77.3-91.6)	0 (-10.67 - 10.67)	>0.9999	71; 79.8 (70.3-86.8)	-6.09 (-17.54 - 5.57)	0.3261	6.09 (-5.57 - 17.54)	0.3261
	4	92; 100 (96.0-100)	92; 100 (96.0-100)	0 (-3.07 - 3.07)	>0.9999	89; 100 (95.9-100)	0 (-3.16 - 3.09)	>0.9999	0 (-3.09 - 3.16)	>0.9999
	5	92; 100 (96.0-100)	90; 97.8 (92.4-99.4)	-2.17 (-6.44 - 2.18)	0.4973	89; 100 (95.9-100)	0 (-3.16 - 3.09)	>0.9999	-2.17 (-6.44 - 2.25)	0.4974

	6A	92; 100 (96.0-100)	89; 96.7 (90.8-98.9)	-3.26 (-7.99 - 1.6)	0.0008	86; 96.6 (90.6-98.8)	-3.37 (-8.25 - 1.59)	0.1168	0.11 (-6 - 6.29)	>0.9999
	6B	91; 98.9 (94.1-99.8)	81; 88.0 (79.8-93.2)	-10.87 (-18.34 to - 2.94)	0.0005	84; 94.4 (87.5-97.6)	-4.53 (-10.62 - 1.69)	0.1138	-6.34 (-15.03 - 2.69)	0.1903
	7F	92; 100 (96.0-100)	91; 98.9 (94.1-99.8)	-1.09 (-4.81 - 2.69)	<0.0001	89; 100 (95.9-100)	0 (-3.16 - 3.09)	>0.9999	-1.09 (-4.82 - 2.76)	>0.9999
	9V	90; 97.8 (92.4-99.4)	92; 100 (96.0-100)	2.17 (-2.18 - 6.44)	0.4402	89; 100 (95.9-100)	2.17 (-2.25 - 6.44)	0.4974	0 (-3.09 - 3.16)	>0.9999
	14	92; 100 (96.0-100)	87; 94.6 (87.9-97.7)	-5.43 (-10.93 - 0.3)	0.0980	89; 100 (95.9-100)	0 (-3.16 - 3.09)	>0.9999	-5.43 (-10.93 - 0.36)	0.0592
	18C	91; 98.9 (94.1-99.8)	89; 96.7 (90.8-98.9)	-2.17 (-7.38 - 3.13)	<0.0001	88; 98.9 (93.9-99.8)	-0.04 (-4.46 - 4.32)	>0.9999	-2.14 (-7.37 - 3.26)	0.6210
	19A	92; 100 (96.0-100)	92; 100 (96.0-100)	0 (-3.07 - 3.07)	0.1468	89; 100 (95.9-100)	0 (-3.16 - 3.09)	>0.9999	0 (-3.09 - 3.16)	>0.9999
	19F	92; 100 (96.0-100)	90; 97.8 (92.4-99.4)	-2.17 (-6.44 - 2.18)	0.5455	87; 97.8 (92.2-99.4)	-2.25 (-6.65 - 2.18)	0.2404	0.07 (-5.25 - 5.46)	>0.9999
	23F	90; 97.8 (92.4-99.4)	88; 95.7 (89.3-98.3)	-2.17 (-8.17 - 3.91)	0.0448	88; 98.9 (93.9-99.8)	1.05 (-3.89 - 5.87)	>0.9999	-3.22 (-8.83 - 2.58)	0.3683

[a]GMC (96% CI) or number over 0.35μg/ml threshold; percentage over 0.35μg/ml threshold (96% CI)

[b]Comparison between 6wk+1-PCV13 or 14wk+1-PCV13 and 2+1-PCV13; GMC compared by ratio between 1+1 / 2+1; percentages compared by difference in percentages above specific threshold between 1+1 and 2+1.

[c]Comparison between 14wk+1-PCV13 and 6wk+1-PCV13; GMC compared by ratio between 14wk/6wk; percentages compared by difference in percentages above specific threshold between 14wk and 6wk (14wk-6wk).

Noninferiority criterion: When comparing GMCs, non-inferiority is shown when the lower limit of the 96% CI of the ratio of GMCs is >0.5 for 10 of the 13 serotypes. When comparing percentages, non-inferiority is shown when the lower limit of the 96% CI of the risk difference is >-10% 10 of the 13 serotypes. .

Supplementary Table S2: Immune responses one-month following the booster dose of 10-valent pneumococcal conjugate vaccine (PCV10) in children who received a single dose at six (6w+1-PCV10) or 14 (14w+1-PCV10) weeks of age, and infants who received two doses at 6 and 14 weeks (2+1-PCV10) of age.

Measurement	Serotype	2+1-PCV10 n=90[a]	6w+1-PCV10 n=93[a]	Compare6wk+1-PCV10 to 2+1-PCV10[b]	p-value[b]	14w+1-PCV10 n=86[a]	Compare14wk+1-PCV10 to 2+1-PCV13[b]	p-value[b]	Comparing 14w+1-PCV10 to 6w+1-PCV10[c]	p-value[c]
GMC	1	5.6 (4.4-7.1)	6.2 (5.0-7.7)	1.1 (0.8-1.53)	0.5328	6 (4.9-7.4)	1.07 (0.78-1.48)	0.6596	0.97 (0.71-1.32)	0.8382
	3*	0.09 (0.07-0.12)	0.12 (0.09-0.15)	1.3 (0.93-1.82)	0.1117	0.13 (0.1-0.16)	1.37 (0.97-1.93)	0.0641	1.05 (0.74-1.5)	0.7681
	4	4.7 (3.7-5.8)	6.8 (5.5-8.4)	1.46 (1.06-2)	0.0140	7.1 (5.8-8.7)	1.52 (1.11-2.09)	0.0068	1.04 (0.77-1.41)	0.7801
	5	3.0 (2.4-3.7)	3.7 (2.8-4.7)	1.23 (0.86-1.77)	0.2280	4.7 (3.9-5.7)	1.58 (1.16-2.16)	0.0026	1.28 (0.91-1.8)	0.1307
	6A*	0.42 (0.31-0.57)	0.33 (0.25-0.42)	0.78 (0.51-1.18)	0.2134	0.38 (0.29-0.48)	0.9 (0.6-1.36)	0.5985	1.16 (0.8-1.68)	0.4197
	6B	5.7 (4.6-7)	4.6 (3.7-5.8)	0.81 (0.59-1.11)	0.1726	4.7 (3.7-5.8)	0.82 (0.6-1.12)	0.1944	1.01 (0.72-1.41)	0.9540
	7F	3.8 (3.2-4.5)	3.6 (3.0-4.2)	0.93 (0.73-1.19)	0.5527	3.8 (3.3-4.4)	1 (0.8-1.25)	0.9836	1.07 (0.85-1.35)	0.5467
	9V	3.9 (3.2-4.8)	3.8 (3.1-4.6)	0.95 (0.71-1.27)	0.7246	3.4 (2.9-4)	0.86 (0.66-1.12)	0.2488	0.91 (0.7-1.18)	0.4407
	14	5.9 (4.2-8.2)	5.7 (4.2-7.9)	0.98 (0.6-1.58)	0.9177	7.4 (5.8-9.5)	1.26 (0.82-1.95)	0.2637	1.29 (0.84-1.99)	0.2124
	18C	3.9 (3.3-4.7)	2.9 (2.5-3.5)	0.75 (0.57-0.97)	0.0224	3.4 (2.7-4.2)	0.86 (0.64-1.15)	0.2857	1.15 (0.87-1.53)	0.3065
	19A*	0.50 (0.37-0.70)	0.58 (0.43-0.79)	1.15 (0.72-1.83)	0.5334	0.64 (0.46-0.88)	1.27 (0.79-2.04)	0.3059	1.10 (0.69-1.76)	0.6692
	19F	4.9 (4.0-6.1)	7.4 (5.5-9.9)	1.51 (1.03-2.2)	0.0266	4.7 (3.3-6.5)	0.95 (0.62-1.44)	0.7880	0.63 (0.4-1)	0.0396
	23F	3.3 (2.8-3.9)	2.6 (2.1-3.2)	0.79 (0.59-1.06)	0.0945	3.5 (2.9-4.3)	1.07 (0.81-1.41)	0.6387	1.35 (0.99-1.84)	0.0497
>=0.35	1	89; 98.9 (94.0-99.8)	93; 100 (96.0-100)	1.11 (-2.67 - 4.91)	0.4918	85; 98.8 (93.7-99.8)	-0.05 (-4.61 - 4.42)	>0.9999	1.16 (-2.69 - 5.13)	0.4804
	3*	6; 6.7 (3.1-13.8)	15; 16.1 (10.0-24.9)	9.46 (-0.48 - 18.95)	0.0623	13; 15.1 (9.1-24.2)	8.45 (-1.52 - 18.12)	0.0900	1.01 (-10.31 - 12.17)	>0.9999
	4	89; 98.9 (94.0-99.8)	92; 98.9 (94.2-99.8)	0.04 (-4.28 - 4.42)	>0.9999	85; 98.8 (93.7-99.8)	-0.05 (-4.61 - 4.42)	>0.9999	0.09 (-4.28 - 4.62)	>0.9999
	5	87; 96.7 (90.7-98.9)	89; 95.7 (89.5-98.3)	-0.97 (-7.33 - 5.5)	>0.9999	86; 100 (95.7-100)	3.33 (-1.73 - 8.16)	0.2461	-4.30 (-9.37 - 1.12)	0.1219

	6A*	42; 46.7 (36.7-56.9)	39; 41.9 (32.4-52.1)	-4.73 (-19.55 - 10.28)	0.5537	41; 47.7 (37.4-58.1)	1.01 (-14.3 - 16.28)	>0.9999	-5.74 (-20.72 - 9.47)	0.4558
	6B	88; 97.8 (92.3-99.4)	89; 95.7 (89.5-98.3)	-2.08 (-8.05 - 4.05)	0.6825	83; 96.5 (90.2-98.8)	-1.27 (-7.22 - 4.65)	0.6769	-0.81 (-7.27 - 5.83)	>0.9999
	7F	90; 100 (95.9-100)	93; 100 (96.0-100)	0 (-3.06 - 3.13)	>0.9999	86; 100 (95.7-100)	0 (-3.26 - 3.16)	>0.9999	0 (-3.08 - 3.25)	>0.9999
	9V	87; 96.7 (90.7-98.9)	92; 98.9 (94.2-99.8)	2.26 (-3.07 - 7.55)	0.3627	85; 98.8 (93.7-99.8)	2.17 (-3.38 - 7.53)	0.6211	0.09 (-4.28 - 4.62)	>0.9999
	14	83; 92.2 (84.8-96.2)	84; 90.3 (82.6-94.8)	-1.9 (-10.67 - 7.01)	0.7949	83; 96.5 (90.2-98.8)	4.29 (-3.41 - 11.71)	0.3307	-6.19 (-13.89 - 1.93)	0.1361
	18C	88; 97.8 (92.3-99.4)	92; 98.9 (94.2-99.8)	1.15 (-3.7 - 6.01)	0.6168	85; 98.8 (93.7-99.8)	1.06 (-4.02 - 6)	>0.9999	0.09 (-4.28 - 4.62)	>0.9999
	19A*	46; 51.1 (41.0-61.2)	57; 61.3 (51.1-70.6)	10.18 (-4.87 - 24.8)	0.1819	55; 64 (53.4-73.3)	12.84 (-2.47 - 27.57)	0.0951	-2.66 (-17.3 - 12.13)	0.7585
	19F	89; 98.9 (94.0-99.8)	90; 96.8 (90.9-98.9)	-2.11 (-7.3 - 3.22)	0.6211	76; 88.4 (79.9-93.6)	-10.52 (-18.21 to -2.44)	0.0042	8.4 (-0.1 - 16.68)	0.0422
	23F	89; 98.9 (94.0-99.8)	89; 95.7 (89.5-98.3)	-3.19 (-8.74 - 2.56)	0.3684	84; 97.7 (91.9-99.4)	-1.21 (-6.29 - 3.82)	0.6144	-1.98 (-8.01 - 4.3)	0.6836

[a]GMC (96% CI) or number over 0.35µg/ml threshold; percentage over 0.35µg/ml threshold (96% CI)

[b]Comparison between 6wk+1-PCV10 or 14wk+1-PCV10 and 2+1-PCV10; GMC compared by ratio between 1+1 / 2+1; percentages compared by difference in percentages above specific threshold between 1+1 and 2+1.

[c]Comparison between 14wk+1-PCV10 and 6wk+1-PCV10; GMC compared by ratio between 14wk/6wk; percentages compared by difference in percentages above specific threshold between 14wk and 6wk (14wk-6wk).

Noninferiority criterion: When comparing GMCs, non-inferiority is shown when the lower limit of the 96% CI of the ratio of GMCs is >0.5 for 8 of the 10 vaccine serotypes. When comparing percentages, non-inferiority is shown when the lower limit of the 96% CI of the risk difference is >-10% for 8 of the 10 vaccine serotypes.

*Serotypes not included in PCV10 formulation

Supplementary Table S3: Opsonophagocytic activity assay (OPA) titers one-month following the booster dose of 13-valent (PCV13) or 10-valent pneumococcal conjugate vaccine (PCV10) in children in children who received a single dose at six (6w+1-PCV) or 14 (14w+1-PCV) weeks of age, and infants who received two doses at 6 and 14 weeks (2+1-PCV).

	13-valent PCV				10-valent PCV			
Measurement	Serotpye	6w+1-PCV13[a,b] n=20	14w+1-PCV13[a,b] n=19	2+1-PCV13[a,b] n=20	Serotpye	6w+1-PCV10[a,b] n=20	14w+1-PCV10[a,b] n=19	2+1-PCV10[a,b] n=19
GMT	1	169 (85-338)	604 (351-1041)	212 (117-383)	1	158 (72-345) n=18	267 (164-434)	196 (88-438) n=18
	3	157 (99-249)	75 (46-122)	93 (63-137)	3*	4 (4-5)	4 (4-4) n=17	4 (4-4)
	4	3026 (1883-4864)	3019 (2003-4550) n=18	2033 (1273-3245)	4	1140 (684-1900) n=18	1508 (1019-2231) n=17	993 (651-1515) n=15
	5	325 (200-528)	1003 (592-1698)	668 (401-1113)	5	401 (192-836)	1127 (714-1779)	645 (368-1132)
	6A	2930 (1630-5267) n=19	3656 (2256-5924) n=18	6064 (4097-8976)	6A*	25 (8-86) n=19	62 (16-242) n=18	35 (9-129) n=18
	6B	906 (377-2180) n=19	1636 (858-3121) n=18	2213 (1537-3188)	6B	611 (359-1041)	941 (626-1414) n=17	722 (351-1489) n=17
	7F	3547 (2349-5356)	3927 (2444-6310)	10481 (6926-15862)	7F	1346 (1036-1749) n=19	2016 (1381-2943)	1828 (1272-2626)
	9V	2082 (1316-3293) n=19	1586 (809-3107)	3070 (1983-4752)	9V	368 (197-687)	870 (552-1371)	550 (289-1048)
	14	4292 (2281-8076)	2112 (1184-3769) n=18	2496 (1785-3490)	14	1247 (577-2697) n=19	1445 (928-2249) n=17	1245 (521-2976) n=18
	18C	1554 (1010-2391)	1406 (899-2201)	3256 (1923-5512)	18C	2002 (1304-3075)	2094 (936-4684)	2626 (1693-4073)
	19A	1581 (868-2878)	1073 (571-2018)	1765 (1045-2982)	19A*	55 (21-141) n=19	40 (13-128) n=18	30 (11-78)
	19F	1222 (414-3609)	1520 (679-3403)	1696 (1094-2629)	19F	795 (340-1861)	469 (142-1556)	1058 (765-1463)
	23F	3325 (1379-8017)	3762 (1910-7408) n=18	12328 (6849-22189)	23F	716 (332-1543)	1068 (749-1521) n=18	991 (687-1430)
>=8	1	19; 95.0 (76.4-99.1)	19; 100 (83.2-100)	20; 100 (83.9-100)	1	17; 94.4 (74.2-99) n=18	19; 100 (83.2-100)	17; 94.4 (74.2-99) n=18
>=8	3	20; 100 (83.9-100)	19; 100 (83.2-100)	20; 100 (83.9-100)	3*	2; 10.0 (2.8-30.1)	0; 0 (0-18.4) n=17	0; 0 (0 -16.8)
>=10	4	20; 100 (83.9-100)	18; 100 (82.4-100) n=18	20; 100 (83.9-100)	4	18; 100 (82.4-100) n=18	17; 100 (81.6-100) n=17	15; 100 (79.6-100) n=15
>=8	5	20; 100 (83.9-100)	19; 100 (83.2-100)	20; 100 (83.9-100)	5	20; 100 (83.9-100)	19; 100 (83.2-100)	19; 100 (83.2-100)
>=8	6A	19; 100 (83.2-100) n=19	18; 100 (82.4-100) n=18	20; 100 (83.9-100)	6A*	9; 47.4 (27.3-68.3) n=19	11; 61.1 (38.6-79.7) n=18	8; 44.4 (24.6-66.3) n=18

>=15	6B	18; 94.7 (75.4-99.1) n=19	18; 100 (82.4-100) n=18	20; 100 (83.9-100)	6B	19; 95.0 (76.4-99.1)	17; 100 (81.6-100) n=17	16; 94.1 (73.0-99) n=17
>=8	7F	20; 100 (83.9-100)	19; 100 (83.2-100)	20; 100 (83.9-100)	7F	19; 100 (83.2-100) n=19	19; 100 (83.2-100)	19; 100 (83.2-100)
>=20	9V	19; 100 (83.2-100) n=19	19; 100 (83.2-100)	20; 100 (83.9-100)	9V	19; 95.0 (76.4-99.1)	19; 100 (83.2-100)	18; 94.7 (75.4-99.1)
>=8	14	20; 100 (83.9-100)	18; 100 (82.4-100) n=18	20; 100 (83.9-100)	14	18; 94.7 (75.4-99.1) n=19	17; 100 (81.6-100) n=17	18; 100 (82.4-100) n=18
>=8	18C	20; 100 (83.9-100)	19; 100 (83.2-100)	20; 100 (83.9-100)	18C	20; 100 (83.9-100)	18; 94.7 (75.4-99.1)	19; 100 (83.2-100)
>=8	19A	20; 100 (83.9-100)	19; 100 (83.2-100)	20; 100 (83.9-100)	19A*	14; 73.7 (51.2-88.2) n=19	12; 66.7 (43.7-83.7) n=18	12; 63.2 (41.0-80.9)
>=8	19F	18; 90 (69.9-97.2)	18; 94.7 (75.4-99.1)	20; 100 (83.9-100)	19F	19; 95.0 (76.4-99.1)	17; 89.5 (68.6-97.1)	19; 100 (83.2-100)
>=19	23F	19; 95 (76.4-99.1)	18; 100 (82.4-100) n=18	20; 100 (83.9-100)	23F	18; 90.0 (69.9-97.2)	18; 100 (82.4-100) n=18	19; 100 (83.2-100)

[a]number over specified threshold; percentage over specified threshold (96% CI)

[b]number of samples analysed is noted with "n=" if not all samples were analysed for that serotype in that group.

*Serotypes not included in PCV10 formulation

Supplementary Table S4: Immune responses following the post-primary series of 13-valent pneumococcal conjugate vaccine (PCV13) in children receiving a single dose at six (6w+1-PCV13) or 14 (14w+1-PCV13) weeks of age, and infants receiving two doses at 6 and 14 weeks of age (2+1-PCV13).

Measurement	Serotype	2+1-PCV13 n=95[a]	6w+1-PCV13 n=93[a]	Compared 6wk+1-PCV13 to 2+1-PCV13[b]	p-value[b]	14w+1-PCV13 n=92[a]	Compare 14wk+1-PCV13 to 2+1-PCV13[b]	p-value[b]	Comparing 14w+1-PCV13 to 6w+1-PCV13[c]	p-value[c]
GMC	1	2.7 (2.2-3.3)	0.70 (0.56-0.88)	0.26 (0.19-0.36)	<0.0001	1.4 (1.1-1.7)	0.5 (0.37-0.68)	<0.0001	1.93 (1.41-2.64)	<0.0001
	3	0.60 (0.52-0.68)	0.63 (0.54-0.73)	1.05 (0.85-1.31)	0.6174	0.96 (0.83-1.11)	1.61 (1.31-1.98)	<0.0001	1.53 (1.22-1.91)	0.0001
	4	1.9 (1.5-2.4)	0.24 (0.19-0.30)	0.13 (0.09-0.18)	<0.0001	0.58 (0.45-0.74)	0.3 (0.21-0.43)	<0.0001	2.41 (1.7-3.41)	<0.0001
	5	1.5 (1.2-1.8)	0.32 (0.25-0.41)	0.22 (0.16-0.31)	<0.0001	0.53 (0.41-0.68)	0.36 (0.26-0.51)	<0.0001	1.64 (1.14-2.35)	0.0055
	6A	1.4 (1.0-1.8)	0.14 (0.12-0.17)	0.1 (0.07-0.15)	<0.0001	0.13 (0.11-0.15)	0.1 (0.07-0.14)	<0.0001	0.92 (0.72-1.17)	0.4820
	6B	0.52 (0.38-0.70)	0.10 (0.08-0.11)	0.19 (0.13-0.27)	<0.0001	0.08 (0.07-0.09)	0.15 (0.11-0.22)	<0.0001	0.82 (0.66-1.03)	0.0772
	7F	3.7 (3.0-4.6)	0.36 (0.28-0.46)	0.1 (0.07-0.13)	<0.0001	1 (0.8-1.3)	0.28 (0.2-0.4)	<0.0001	2.92 (2.04-4.19)	<0.0001
	9V	1.5 (1.1-2.0)	0.20 (0.17-0.25)	0.14 (0.09-0.2)	<0.0001	0.3 (0.22-0.4)	0.2 (0.13-0.3)	<0.0001	1.46 (1.02-2.09)	0.0292
	14	2.1 (1.5-3.0)	0.58 (0.46-0.74)	0.27 (0.17-0.43)	<0.0001	0.66 (0.52-0.84)	0.31 (0.2-0.49)	<0.0001	1.14 (0.8-1.63)	0.4520
	18C	1.3 (1-1.6.0)	0.33 (0.27-0.40)	0.25 (0.19-0.35)	<0.0001	0.37 (0.3-0.44)	0.28 (0.21-0.39)	<0.0001	1.11 (0.85-1.46)	0.4253
	19A	2.1 (1.7-2.7)	0.43 (0.36-0.52)	0.21 (0.15-0.28)	<0.0001	0.45 (0.37-0.55)	0.21 (0.16-0.29)	<0.0001	1.03 (0.78-1.36)	0.8233
	19F	3.8 (3-4.7.0)	0.82 (0.71-0.94)	0.22 (0.16-0.29)	<0.0001	1 (0.9-1.3)	0.28 (0.2-0.38)	<0.0001	1.27 (1-1.62)	0.0407
	23F	0.94 (0.70-1.27)	0.09 (0.07-0.10)	0.09 (0.06-0.13)	<0.0001	0.09 (0.07-0.11)	0.1 (0.07-0.14)	<0.0001	1.04 (0.79-1.38)	0.7615
>=0.35	1	91; 95.8 (89.7-98.4)	70; 75.3 (65.6-82.9)	-20.52 (-30.36 to -9.86)	<0.0001	80; 87 (78.6-92.4)	-8.83 (-17.32 to -0.03)	0.0373	-11.69 (-23.15 - 0.28)	0.0596
	3	80; 84.2 (75.6-90.2)	71; 76.3 (66.8-83.8)	-7.87 (-19.61 - 4.17)	0.2014	83; 90.2 (82.4-94.8)	6.01 (-4.27 - 15.98)	0.2758	-13.87 (-24.71 to -2.43)	0.0172
	4	87; 91.6 (84.3-95.7)	33; 35.5 (26.5-45.6)	-56.1 (-66.71 to -43.16)	<0.0001	62; 67.4 (57.3-76.1)	-24.19 (-35.35 to -12.05)	<0.0001	-31.91 (-45.42 to -17.05)	<0.0001
	5	87; 91.6 (84.3-95.7)	43; 46.2 (36.5-56.3)	-45.34 (-56.53 to -32.28)	<0.0001	57; 62 (51.7-71.2)	-29.62 (-40.96 to -17.08)	<0.0001	-15.72 (-30.1 to -0.67)	0.0390

	6A	76; 80.0 (70.9-86.8)	10; 10.8 (5.9-18.7)	-69.25 (-78.6 to -57)	<0.0001	11; 12 (6.8-20.2)	-68.04 (-77.62 to -55.61)	<0.0001	-1.2 (-10.96 - 8.58)	0.8209
	6B	58; 61.1 (51.0-70.2)	6; 6.5 (3.0-13.4)	-54.6 (-65.03 to -41.88)	<0.0001	2; 2.2 (0.6-7.6)	-58.88 (-68.47 to -46.79)	<0.0001	4.28 (-2.47 - 10.82)	0.2783
	7F	92; 96.8 (91.1-98.9)	50; 53.8 (43.7-63.5)	-43.08 (-53.49 to -30.9)	<0.0001	76; 82.6 (73.6-89)	-14.23 (-23.11 to -4.81)	0.0013	-28.85 (-41.53 to -14.93)	<0.0001
	9V	81; 85.3 (76.8-91.0)	24; 25.8 (18.0-35.5)	-59.46 (-70.18 to -46.26)	<0.0001	37; 40.2 (30.8-50.4)	-45.05 (-56.95 to -31.27)	<0.0001	-14.41 (-28.04 to -0.18)	0.0427
	14	80; 84.2 (75.6-90.2)	65; 69.9 (59.9-78.3)	-14.32 (-26.44 to -1.62)	0.0239	68; 73.9 (64.1-81.8)	-10.3 (-22.25 - 2.04)	0.1052	-4.02 (-17.41 - 9.55)	0.6243
	18C	79; 83.2 (74.4-89.4)	47; 50.5 (40.6-60.5)	-32.62 (-45.13 to -18.76)	<0.0001	44; 47.8 (37.9-57.9)	-35.33 (-47.82 to -21.38)	<0.0001	2.71 (-12.28 - 17.59)	0.7693
	19A	88; 92.6 (85.6-96.4)	52; 55.9 (45.8-65.6)	-36.72 (-47.9 to -24.03)	<0.0001	53; 57.6 (47.4-67.2)	-35.02 (-46.25 to -22.36)	<0.0001	-1.69 (-16.46 - 13.15)	0.8823
	19F	92; 96.8 (91.1-98.9)	85; 91.4 (83.9-95.6)	-5.44 (-12.78 - 2.08)	0.1310	85; 92.4 (85.1-96.3)	-4.45 (-11.61 - 2.83)	0.2079	-0.99 (-9.51 - 7.58)	>0.9999
	23F	72; 75.8 (66.3-83.3)	9; 9.7 (5.2-17.4)	-66.11 (-75.81 to -53.65)	<0.0001	9; 9.8 (5.2-17.6)	-66.01 (-75.74 to -53.5)	<0.0001	-0.11 (-9.3 - 9.08)	>0.9999

[a]GMC (96% CI) or number over 0.35µg/ml threshold; percentage over 0.35µg/ml threshold (96% CI)

[b]Comparison between 6wk+1-PCV13 14wk+1-PCV13 and 2+1-PCV13; GMC compared by ratio between 1+1 / 2+1; percentages compared by difference in percentages above specific threshold between 1+1 and 2+1.

[c]Comparison between 14wk+1-PCV13 and 6wk+1-PCV13; GMC compared by ratio between 14wk/6wk; percentages compared by difference in percentages above specific threshold between 14wk and 6wk (14wk-6wk).

Noninferiority criterion: When comparing GMCs, non-inferiority is shown when the lower limit of the 96% CI of the ratio of GMCs is >0.5 for 10 of the 13 serotypes. When comparing percentages, non-inferiority is shown when the lower limit of the 96% CI of the risk difference is >-10% for 10 of the 13 serotypes.

Supplementary Table S5: Immune responses following the post-primary series of 10-valent pneumococcal conjugate vaccine (PCV10) in children receiving a single dose at six (6w+1-PCV10) or 14 (14w+1-PCV10) weeks of age, and infants receiving two doses at 6 and 14 weeks of age.

Measurement	Serotype	2+1-PCV10 n=93[a]	6w+1-PCV10 n=94[a]	Compare6wk+1-PCV10 to 2+1-PCV10[b]	p-value[b]	14w+1-PCV10 n=91[a]	Compare14wk+1-PCV10 to 2+1-PCV13[b]	p-value[b]	Comparing 14w+1-PCV10 to 6w+1-PCV10[c]	p-value[c]
GMC	1	2.6 (2.2-3.0)	1.9 (1.5-2.4)	0.73 (0.54-0.98)	0.0284	1.7 (1.4-2.1)	0.68 (0.53-0.89)	0.0029	0.94 (0.67-1.3)	0.6790
	3*	0.08 (0.06-0.09)	0.13 (0.11-0.15)	1.68 (1.3-2.17)	<0.0001	0.08 (0.07-0.1)	1.07 (0.81-1.41)	0.6143	0.64 (0.49-0.82)	0.0003
	4	2.5 (2-3.1.0)	0.40 (0.30-0.52)	0.16 (0.11-0.22)	<0.0001	1.1 (0.8-1.4)	0.42 (0.3-0.59)	<0.0001	2.69 (1.83-3.95)	<0.0001
	5	1.6 (1.3-1.9)	0.90 (0.71-1.15)	0.57 (0.42-0.77)	0.0002	1 (0.8-1.3)	0.65 (0.48-0.87)	0.0023	1.14 (0.81-1.61)	0.4172
	6A*	0.13 (0.11-0.15)	0.14 (0.12-0.17)	1.11 (0.87-1.42)	0.3731	0.11 (0.1-0.13)	0.86 (0.68-1.08)	0.1790	0.77 (0.61-0.97)	0.0228
	6B	1.2 (0.9-1.6)	0.14 (0.12-0.18)	0.12 (0.08-0.17)	<0.0001	0.09 (0.08-0.11)	0.08 (0.05-0.11)	<0.0001	0.64 (0.47-0.86)	0.0024
	7F	2.7 (2.2-3.3)	0.48 (0.36-0.63)	0.17 (0.12-0.25)	<0.0001	0.85 (0.66-1.08)	0.31 (0.22-0.43)	<0.0001	1.77 (1.2-2.62)	0.0029
	9V	1.7 (1.4-2.2)	0.36 (0.29-0.46)	0.21 (0.15-0.3)	<0.0001	0.34 (0.27-0.43)	0.2 (0.14-0.28)	<0.0001	0.93 (0.66-1.31)	0.6745
	14	4.4 (3.3-5.7)	0.79 (0.62-1.00)	0.18 (0.13-0.26)	<0.0001	0.46 (0.37-0.57)	0.11 (0.07-0.15)	<0.0001	0.58 (0.42-0.81)	0.0009
	18C	0.87 (0.68-1.10)	0.22 (0.18-0.26)	0.25 (0.18-0.34)	<0.0001	0.31 (0.26-0.38)	0.36 (0.26-0.49)	<0.0001	1.43 (1.09-1.88)	0.0067
	19A*	0.19 (0.16-0.23)	0.33 (0.27-0.40)	1.74 (1.32-2.28)	<0.0001	0.18 (0.15-0.22)	0.95 (0.73-1.24)	0.7110	0.55 (0.42-0.72)	<0.0001
	19F	2.0 (1.6-2.5)	0.81 (0.68-0.97)	0.4 (0.3-0.53)	<0.0001	0.61 (0.5-0.74)	0.3 (0.22-0.41)	<0.0001	0.76 (0.58-0.99)	0.0341
	23F	0.94 (0.74-1.19)	0.11 (0.09-0.13)	0.12 (0.08-0.16)	<0.0001	0.09 (0.07-0.11)	0.1 (0.07-0.13)	<0.0001	0.83 (0.61-1.12)	0.1976
>=0.35	1	93; 100 (96.0-100)	86; 91.5 (84.1-95.6)	-8.51 (-14.8 to -1.85)	0.0067	83; 91.2 (83.6-95.5)	-8.79 (-15.28 to -1.97)	0.0030	0.28 (-8.47 - 9.08)	>0.9999
	3*	4; 4.3 (1.7-10.5)	9; 9.6 (5.1-17.2)	5.27 (-2.79 - 13.1)	0.2496	5; 5.5 (2.4-12.2)	1.19 (-5.85 - 8.22)	0.7457	4.08 (-4.3 - 12.23)	0.4062
	4	90; 96.8 (90.9-98.9)	49; 52.1 (42.1-61.9)	-44.65 (-55 to -32.41)	<0.0001	76; 83.5 (74.6-89.7)	-13.26 (-22.08 to -3.91)	0.0026	-31.39 (-43.91 to -17.51)	<0.0001
	5	88; 94.6 (88.0-97.7)	71; 75.5 (66.0-83.1)	-19.09 (-29.11 to -8.26)	0.0003	78; 85.7 (77.1-91.5)	-8.91 (-17.92 - 0.44)	0.0492	-10.18 (-21.79 - 1.9)	0.0957

	6A*	13; 14.0 (8.4-22.5)	14; 14.9 (9.1-23.5)	0.92 (-9.78 - 11.55)	>0.9999	10; 11 (6.1-19.1)	-2.99 (-13.06 - 7.24)	0.6570	3.9 (-6.46 - 14.05)	0.5138
	6B	71; 76.3 (66.8-83.8)	18; 19.1 (12.5-28.3)	-57.2 (-68.29 to -43.7)	<0.0001	11; 12.1 (6.9-20.4)	-64.26 (-74.39 to -51.38)	<0.0001	7.06 (-4.1 - 17.88)	0.2267
	7F	90; 96.8 (90.9-98.9)	61; 64.9 (54.8-73.8)	-31.88 (-42.09 to -20.32)	<0.0001	71; 78 (68.5-85.3)	-18.75 (-28.23 to -8.51)	<0.0001	-13.13 (-26.24 - 0.57)	0.0527
	9V	88; 94.6 (88.0-97.7)	41; 43.6 (34.0-53.7)	-51.01 (-61.53 to -38.34)	<0.0001	46; 50.5 (40.5-60.6)	-44.07 (-54.96 to -31.33)	<0.0001	-6.93 (-21.67 - 8.1)	0.3786
	14	89; 95.7 (89.5-98.3)	69; 73.4 (63.7-81.3)	-22.29 (-32.26 to -11.38)	<0.0001	59; 64.8 (54.6-73.9)	-30.86 (-41.44 to -19)	<0.0001	8.57 (-5.41 - 22.21)	0.2649
	18C	76; 81.7 (72.7-88.3)	24; 25.5 (17.8-35.2)	-56.19 (-67.37 to -42.65)	<0.0001	41; 45.1 (35.2-55.3)	-36.67 (-49.33 to -22.46)	<0.0001	-19.52 (-33.15 to -5.09)	0.0059
	19A*	22; 23.7 (16.2-33.2)	42; 44.7 (35.0-54.7)	21.02 (6.79 - 34.37)	0.0033	20; 22 (14.7-31.5)	-1.68 (-14.31 - 11.05)	0.8612	22.7 (8.5 - 35.92)	0.0011
	19F	91; 97.8 (92.5-99.4)	84; 89.4 (81.5-94.1)	-8.49 (-15.93 to -0.67)	0.0327	69; 75.8 (66.1-83.5)	-22.03 (-31.47 to -11.67)	<0.0001	13.54 (1.92 - 24.63)	0.0192
	23F	71; 76.3 (66.8-83.8)	13; 13.8 (8.3-22.2)	-62.51 (-72.88 to -49.54)	<0.0001	9; 9.9 (5.3-17.7)	-66.45 (-76.22 to -53.86)	<0.0001	3.94 (-6.08 - 13.74)	0.4978

[a]GMC (96% CI) or number over 0.35µg/ml threshold; percentage over 0.35µg/ml threshold (96% CI)

[b]Comparison between 6wk+1-PCV10 or 14wk+1-PCV10 and 2+1-PCV10; GMC compared by ratio between 1+1 / 2+1; percentages compared by difference in percentages above specific threshold between 1+1 and 2+1.

[c]Comparison between 14wk+1-PCV10 and 6wk+1-PCV10; GMC compared by ratio between 14wk/6wk; percentages compared by difference in percentages above specific threshold between 14wk and 6wk (14wk-6wk).

Noninferiority criterion: When comparing GMCs, non-inferiority is shown when the lower limit of the 96% CI of the ratio of GMCs is >0.5 for 8 of the 10 vaccine serotypes. When comparing percentages, non-inferiority is shown when the lower limit of the 96% CI of the risk difference is >-10% for 8 of the 10 vaccine serotypes.

*Serotypes not included in PCV10 formulation

Supplementary Table S6: Immune responses prior to the booster dose of 13-valent pneumococcal conjugate vaccine (PCV13) in children receiving a single dose at six (6w+1-PCV-13) or 14 (14w+1-PCV13) weeks of age, and infants receiving two doses at 6 and 14 weeks of age (2+1-PCV13).

Measurement	Serotype	2+1-PCV13 n=76[a]	6w+1-PCV13 n=73[a]	Compare 6wk+1-PCV13 to 2+1-PCV13[b]	p-value[b]	14w+1-PCV13 n=69[a]	Compare 14wk+1-PCV13 to 2+1-PCV13[b]	p-value[b]	Comparing 14w+1-PCV13 to 6w+1-PCV13[c]	p-value[c]
GMC	1	1.2 (1.0-1.5)	0.46 (0.35-0.61)	0.38 (0.27-0.54)	<0.0001	0.85 (0.71-1.01)	0.7 (0.53-0.93)	0.0107	1.84 (1.31-2.6)	0.0003
	3	0.21 (0.17-0.27)	0.16 (0.13-0.21)	0.77 (0.54-1.11)	0.1364	0.24 (0.2-0.3)	1.16 (0.83-1.61)	0.3597	1.5 (1.07-2.11)	0.0136
	4	0.38 (0.30-0.47)	0.07 (0.06-0.09)	0.18 (0.13-0.25)	<0.0001	0.31 (0.24-0.39)	0.81 (0.57-1.15)	0.2144	4.44 (3.17-6.23)	<0.0001
	5	0.49 (0.40-0.61)	0.14 (0.11-0.18)	0.29 (0.21-0.4)	<0.0001	0.45 (0.35-0.57)	0.92 (0.66-1.28)	0.6026	3.17 (2.25-4.46)	<0.0001
	6A	0.66 (0.52-0.84)	0.16 (0.14-0.19)	0.25 (0.18-0.33)	<0.0001	0.3 (0.24-0.39)	0.46 (0.32-0.66)	<0.0001	1.86 (1.38-2.51)	<0.0001
	6B	0.30 (0.24-0.38)	0.10 (0.08-0.11)	0.32 (0.24-0.44)	<0.0001	0.14 (0.11-0.17)	0.46 (0.33-0.63)	<0.0001	1.41 (1.09-1.82)	0.0061
	7F	1.1 (0.9-1.4)	0.29 (0.23-0.36)	0.25 (0.18-0.35)	<0.0001	1.1 (0.9-1.4)	1.01 (0.74-1.38)	0.9364	3.97 (2.85-5.52)	<0.0001
	9V	0.57 (0.44-0.72)	0.12 (0.10-0.14)	0.21 (0.15-0.29)	<0.0001	0.37 (0.3-0.47)	0.66 (0.47-0.94)	0.0146	3.18 (2.34-4.33)	<0.0001
	14	1.7 (1.2-2.4)	0.32 (0.25-0.42)	0.19 (0.12-0.3)	<0.0001	0.85 (0.62-1.17)	0.5 (0.31-0.82)	0.0041	2.63 (1.71-4.04)	<0.0001
	18C	0.36 (0.30-0.44)	0.14 (0.10-0.18)	0.37 (0.26-0.53)	<0.0001	0.26 (0.21-0.33)	0.73 (0.54-0.99)	0.0327	1.96 (1.36-2.81)	0.0002
	19A	0.45 (0.35-0.56)	0.27 (0.02-0.36)	0.6 (0.41-0.88)	0.0067	0.3 (0.23-0.38)	0.66 (0.46-0.95)	0.0196	1.11 (0.74-1.66)	0.5962
	19F	1.0 (0.8-1.3)	0.57 (0.43-0.75)	0.55 (0.38-0.8)	0.0011	0.66 (0.55-0.79)	0.65 (0.48-0.88)	0.0034	1.16 (0.83-1.64)	0.3610
	23F	0.21 (0.16-0.28)	0.07 (0.05-0.08)	0.31 (0.22-0.44)	<0.0001	0.1 (0.08-0.12)	0.45 (0.32-0.63)	<0.0001	1.45 (1.09-1.93)	0.0083
>=0.35	1	73; 96.1 (89.0-98.6)	43; 58.9 (47.4-69.5)	-37.15 (-48.96 to -23.45)	<0.0001	61; 88.4 (78.8-94)	-7.65 (-17.14 - 2.05)	0.1169	-29.5 (-42.87 to -14.44)	0.0001
	3	18; 23.7 (15.5-34.4)	9; 12.3 (6.6-21.8)	-11.36 (-23.86 - 1.8)	0.0897	24; 34.8 (24.6-46.6)	11.1 (-4.48 - 26.19)	0.1483	-22.45 (-36.04 to -7.72)	0.0025
	4	42; 55.3 (44.1-65.9)	3; 4.1 (1.4-11.4)	-51.15 (-62.53 to -37.06)	<0.0001	30; 43.5 (32.4-55.2)	-11.78 (-28.2 - 5.26)	0.1846	-39.37 (-51.54 to -25.12)	<0.0001
	5	48; 63.2 (51.9-73.1)	13; 17.8 (10.7-28.1)	-45.35 (-58.7 to -29.6)	<0.0001	44; 63.8 (52-74.1)	0.61 (-15.69 - 16.81)	>0.9999	-45.96 (-59.66 to -29.77)	<0.0001

	6A	54; 71.1 (60.0-80.0)	8; 11.0 (5.7-20.2)	-60.09 (-71.62 to -45.4)	<0.0001	30; 43.5 (32.4-55.2)	-27.57 (-42.93 to -10.77)	0.0013	-32.52 (-46 to -17.33)	<0.0001
	6B	37; 48.7 (37.8-59.7)	2; 2.7 (0.8-9.5)	-45.94 (-57.24 to -32.2)	<0.0001	8; 11.6 (6-21.2)	-37.09 (-50.21 to -21.87)	<0.0001	-8.85 (-18.02 - 0.67)	0.0508
	7F	68; 89.5 (80.6-94.6)	28; 38.4 (28.1-49.8)	-51.12 (-63.53 to -36.06)	<0.0001	62; 89.9 (80.5-95)	0.38 (-10.43 - 10.98)	>0.9999	-51.5 (-63.95 to -36.18)	<0.0001
	9V	55; 72.4 (61.4-81.2)	6; 8.2 (3.8-16.8)	-64.15 (-75 to -49.93)	<0.0001	39; 56.5 (44.8-67.6)	-15.85 (-31.45 - 0.53)	0.0560	-48.3 (-60.92 to -33.09)	<0.0001
	14	60; 78.9 (68.5-86.6)	29; 39.7 (29.3-51.2)	-39.22 (-53.28 to -23.13)	<0.0001	50; 72.5 (61-81.6)	-6.48 (-20.95 - 8.2)	0.4381	-32.74 (-47.81 to -15.86)	0.0001
	18C	37; 48.7 (37.8-59.7)	12; 16.4 (9.7-26.6)	-32.25 (-46.07 to -16.7)	<0.0001	27; 39.1 (28.5-50.9)	-9.55 (-25.92 - 7.36)	0.3151	-22.69 (-37.02 to -7.19)	0.0028
	19A	43; 56.6 (45.4-67.1)	22; 30.1 (20.8-41.4)	-26.44 (-41.64 to -9.85)	0.0016	30; 43.5 (32.4-55.2)	-13.1 (-29.45 - 3.96)	0.1357	-13.34 (-29.3 - 3.31)	0.1180
	19F	67; 88.2 (79.0-93.6)	45; 61.6 (50.2-71.9)	-26.51 (-39.77 to -11.92)	0.0003	56; 81.2 (70.4-88.6)	-7 (-19.33 - 5.53)	0.2568	-19.52 (-34.03 to -3.87)	0.0155
	23F	23; 30.3 (21.1-41.3)	2; 2.7 (0.8-9.5)	-27.52 (-38.46 to -15.07)	<0.0001	5; 7.2 (3.1-15.9)	-23.02 (-35.01 to -9.62)	0.0006	-4.51 (-12.67 - 3.77)	0.2653

[a]GMC (96% CI) or number over 0.35µg/ml threshold; percentage over 0.35µg/ml threshold (96% CI)

[b]Comparison between 6wk+1[PCV13 or 14wk+1-PCV13 and 2+1-PCV13; GMC compared by ratio between 1+1 / 2+1; percentages compared by difference in percentages above specific threshold between 1+1 and 2+1.

[c]Comparison between 14wk+1-PCV13 and 6wk+1-PCV13; GMC compared by ratio between 14wk/6wk; percentages compared by difference in percentages above specific threshold between 14wk and 6wk (14wk-6wk).

Noninferiority criterion: When comparing GMCs, non-inferiority is shown when the lower limit of the 96% CI of the ratio of GMCs is >0.5 for 10 of the 13 serotypes. When comparing percentages, non-inferiority is shown when the lower limit of the 96% CI of the risk difference is >-10% for 10 of the 13 serotypes.

[e]number over 0.35µg/ml threshold; percentage over 0.35µg/ml threshold (95% CI)

Supplementary Table S7: Immune responses prior to the booster dose of 10-valent pneumococcal conjugate vaccine (PCV10) in children receiving a single dose at six (6w+1-PCV10) or 14 (14w+1-PCV10) weeks of age, and infants receiving two doses at 6 and 14 weeks of age (2+1-PCV10).

Measurement	Serotype	2+1-PCV10[a] n=73	6w+1-PCV10[a] n=76	Compare 6wk+1-PCV10 to 2+1-PCV10[b]	p-value[b]	14w+1-PCV10[a] n=69	Compare 14wk+1-PCV10 to 2+1-PCV13[b]	p-value[b]	Comparing 14w+1-PCV10 to 6w+1-PCV10[c]	p-value[c]
GMC	1	0.92 (0.75-1.11)	0.67 (0.53-0.85)	0.73 (0.53-1.01)	0.0480	0.95 (0.75-1.2)	1.04 (0.75-1.42)	0.8143	1.42 (0.99-2.01)	0.0432
	3*	0.07 (0.05-0.09)	0.08 (0.07-0.11)	1.25 (0.86-1.81)	0.2198	0.1 (0.07-0.12)	1.43 (0.98-2.1)	0.0537	1.15 (0.8-1.65)	0.4290
	4	0.73 (0.59-0.91)	0.24 (0.19-0.31)	0.33 (0.24-0.46)	<0.0001	0.65 (0.51-0.84)	0.89 (0.64-1.25)	0.4854	2.69 (1.89-3.84)	<0.0001
	5	0.40 (0.32-0.50)	0.23 (0.18-0.30)	0.58 (0.4-0.83)	0.0018	0.34 (0.27-0.42)	0.84 (0.61-1.17)	0.2813	1.46 (1.02-2.09)	0.0301
	6A*	0.21 (0.16-0.26)	0.16 (0.14-0.18)	0.75 (0.57-1)	0.0372	0.2 (0.16-0.25)	0.97 (0.7-1.34)	0.8267	1.29 (1-1.66)	0.0438
	6B	1.2 (1.0-1.6)	0.43 (0.34-0.54)	0.34 (0.24-0.48)	<0.0001	0.78 (0.63-0.96)	0.62 (0.45-0.86)	0.0032	1.82 (1.3-2.55)	0.0003
	7F	0.93 (0.73-1.20)	0.51 (0.39-0.66)	0.54 (0.37-0.79)	0.0010	1 (0.8-1.3)	1.12 (0.79-1.59)	0.5068	2.07 (1.44-2.96)	<0.0001
	9V	0.72 (0.59-0.87)	0.35 (0.28-0.43)	0.48 (0.35-0.65)	<0.0001	0.57 (0.45-0.71)	0.79 (0.58-1.07)	0.1138	1.63 (1.18-2.27)	0.0022
	14	1.5 (1.1-2.1)	0.39 (0.30-0.51)	0.26 (0.17-0.41)	<0.0001	1.1 (0.8-1.4)	0.71 (0.45-1.13)	0.1330	2.69 (1.76-4.11)	<0.0001
	18C	0.41 (0.32-0.53)	0.11 (0.09-0.14)	0.27 (0.18-0.38)	<0.0001	0.24 (0.18-0.3)	0.57 (0.39-0.83)	0.0024	2.16 (1.49-3.13)	<0.0001
	19A*	0.13 (0.11-0.16)	0.13 (0.11-0.16)	1 (0.76-1.31)	0.9755	0.15 (0.12-0.18)	1.11 (0.84-1.47)	0.4488	1.11 (0.85-1.45)	0.4058
	19F	1.2 (0.9-1.6)	0.52 (0.39-0.68)	0.42 (0.28-0.63)	<0.0001	0.75 (0.58-0.98)	0.61 (0.41-0.9)	0.0102	1.45 (0.97-2.17)	0.0545
	23F	0.46 (0.36-0.59)	0.14 (0.11-0.17)	0.3 (0.21-0.43)	<0.0001	0.27 (0.22-0.34)	0.59 (0.41-0.84)	0.0021	1.95 (1.41-2.69)	<0.0001
>=0.35	1	64; 87.7 (78.2-93.4)	55; 72.4 (61.4-81.2)	-15.3 (-28.08 to -1.66)	0.0246	59; 85.5 (75.3-91.9)	-2.16 (-14.11 - 9.79)	0.8070	-13.14 (-26.4 - 0.97)	0.0683
	3*	6; 8.2 (3.8-16.8)	4; 5.3 (2.1-12.8)	-2.96 (-11.87 - 6.02)	0.5278	6; 8.7 (4-17.7)	0.48 (-9.49 - 10.55)	>0.9999	-3.43 (-12.68 - 5.78)	0.5188
	4	60; 82.2 (71.9-89.3)	22; 28.9 (20.0-40.0)	-53.24 (-65.91 to -37.78)	<0.0001	50; 72.5 (61-81.6)	-9.73 (-23.84 - 4.84)	0.2278	-43.52 (-57.6 to -27.09)	<0.0001
	5	42; 57.5 (46.1-68.2)	28; 36.8 (26.9-48.1)	-20.69 (-36.4 to -3.91)	0.0140	33; 47.8 (36.5-59.4)	-9.71 (-26.35 - 7.46)	0.3131	-10.98 (-27.28 - 5.86)	0.2383

	6A*	15; 20.5 (12.9-31.2)	8; 10.5 (5.4-19.4)	-10.02 (-22.02 - 2.44)	0.1137	17; 24.6 (16-36)	4.09 (-10.36 - 18.4)	0.6883	-14.11 (-26.76 to -0.87)	0.0289
	6B	66; 90.4 (81.5-95.3)	47; 61.8 (50.6-71.9)	-28.57 (-41.27 to -14.32)	<0.0001	60; 87 (77-93)	-3.45 (-14.62 - 7.78)	0.6002	-25.11 (-38.51 to -10.24)	0.0006
	7F	62; 84.9 (75.0-91.4)	51; 67.1 (55.9-76.6)	-17.83 (-31.32 to -3.34)	0.0130	61; 88.4 (78.8-94)	3.47 (-8.56 - 15.21)	0.6260	-21.3 (-34.29 to -7.02)	0.0027
	9V	62; 84.9 (75.0-91.4)	38; 50.0 (39.0-61.0)	-34.93 (-48.52 to -19.48)	<0.0001	53; 76.8 (65.6-85.2)	-8.12 (-21.5 - 5.61)	0.2853	-26.81 (-41.66 to -10.46)	0.0011
	14	60; 82.2 (71.9-89.3)	37; 48.7 (37.8-59.7)	-33.51 (-47.46 to -17.77)	<0.0001	58; 84.1 (73.7-90.9)	1.87 (-11.23 - 14.76)	0.8252	-35.37 (-49.16 to -19.6)	<0.0001
	18C	46; 63.0 (51.5-73.2)	10; 13.2 (7.3-22.6)	-49.86 (-62.6 to -34.53)	<0.0001	22; 31.9 (22.1-43.6)	-31.13 (-46.45 to -14.1)	0.0002	-18.73 (-32.28 to -4.31)	0.0088
	19A*	7; 9.6 (4.7-18.5)	10; 13.2 (7.3-22.6)	3.57 (-7.48 - 14.35)	0.6089	12; 17.4 (10.2-28)	7.8 (-4.29 - 19.58)	0.2198	-4.23 (-16.63 - 8.22)	0.4970
	19F	65; 89.0 (79.8-94.3)	46; 60.5 (49.3-70.8)	-28.51 (-41.49 to -14)	<0.0001	53; 76.8 (65.6-85.2)	-12.23 (-24.89 - 1)	0.0724	-16.29 (-31.22 to -0.38)	0.0490
	23F	47; 64.4 (52.9-74.4)	13; 17.1 (10.3-27.1)	-47.28 (-60.52 to -31.59)	<0.0001	27; 39.1 (28.5-50.9)	-25.25 (-41.04 to -8.09)	0.0041	-22.03 (-36.37 to -6.6)	0.0049

[a]GMC (96% CI) or number over 0.35µg/ml threshold; percentage over 0.35µg/ml threshold (96% CI)

[b]Comparison between 6wk+1-PCV10 or 14wk+1-PCV10 and 2+1-PCV10; GMC compared by ratio between 1+1 / 2+1; percentages compared by difference in percentages above specific threshold between 1+1 and 2+1.

[c]Comparison between 14wk+1-PCV10 and 6wk+1-PCV10; GMC compared by ratio between 14wk/6wk; percentages compared by difference in percentages above specific threshold between 14wk and 6wk (14wk-6wk).

Noninferiority criterion: When comparing GMCs, non-inferiority is shown when the lower limit of the 96% CI of the ratio of GMCs is >0.5 for 8 of the 10 vaccine serotypes. When comparing percentages, non-inferiority is shown when the lower limit of the 96% CI of the risk difference is >-10% for 8 of the 10 vaccine serotypes.

*Serotypes not included in PCV10 formulation

Supplementary Table S8: Line listing of serious adverse events

	6w+1-PCV10	6w+1-PCV13	14w+1-PCV10	14w+1-PCV13	2+1-PCV10	2+1-PCV13	Total
Acute gastro-enteritis	0	2	1	2	1	2	8
Adverse event following hexaxim immunization (vomiting)	0	0	0	0	0	1	1
Adverse event following varicella immunization (rash)	0	0	0	0	1	0	1
Anaphylactic reaction to Penicillin	0	0	0	0	1	0	1
Bronchiolitis	4	6	4	2	5	4	25
Dysentery	0	0	0	0	1	0	1
Febrile seizures	1	0	1	0	2	0	4
Fever of unknown origin	0	1	0	0	0	0	1
Herpetic gingivostomatitis	1	0	0	0	0	0	1
Laryngotracheobronchitis and Subglottic Stenosis	1	0	0	0	0	0	1
Lymphadenitis submandibular	0	0	0	0	0	1	1
Pancytopenia	1	0	0	0	0	0	1
Pneumonia	3	4	2	4	4	4	21
Pulmonary tuberculosis	1	0	1	2	0	3	7
Reaction to vaccine	0	0	0	0	1	0	1
Right clavicle fracture	0	0	0	1	0	0	1
Scabies	0	0	1	0	0	0	1
Staphylococcal sepsis	0	0	0	0	0	1	1
Suspected opioid ingestion	1	0	0	0	0	0	1
Suspected septic arthritis	0	0	1	0	0	0	1
Tonsillitis	0	0	0	0	1	0	1
Upper respiratory tract infection	0	0	0	0	0	1	1
Urinary tract infection	0	0	0	0	0	1	1
Viral Pneumonia	1	0	0	0	0	0	1
Total	14	13	11	11	17	18	84

Supplementary Figure 1: Study schedule of vaccination and evaluation of immune responses in infants vaccinated with either one or two doses of a primary series, followed by a booster dose, of 10-valent (PCV10) or 13-valent (PCV13) pneumococcal conjugate vaccine.

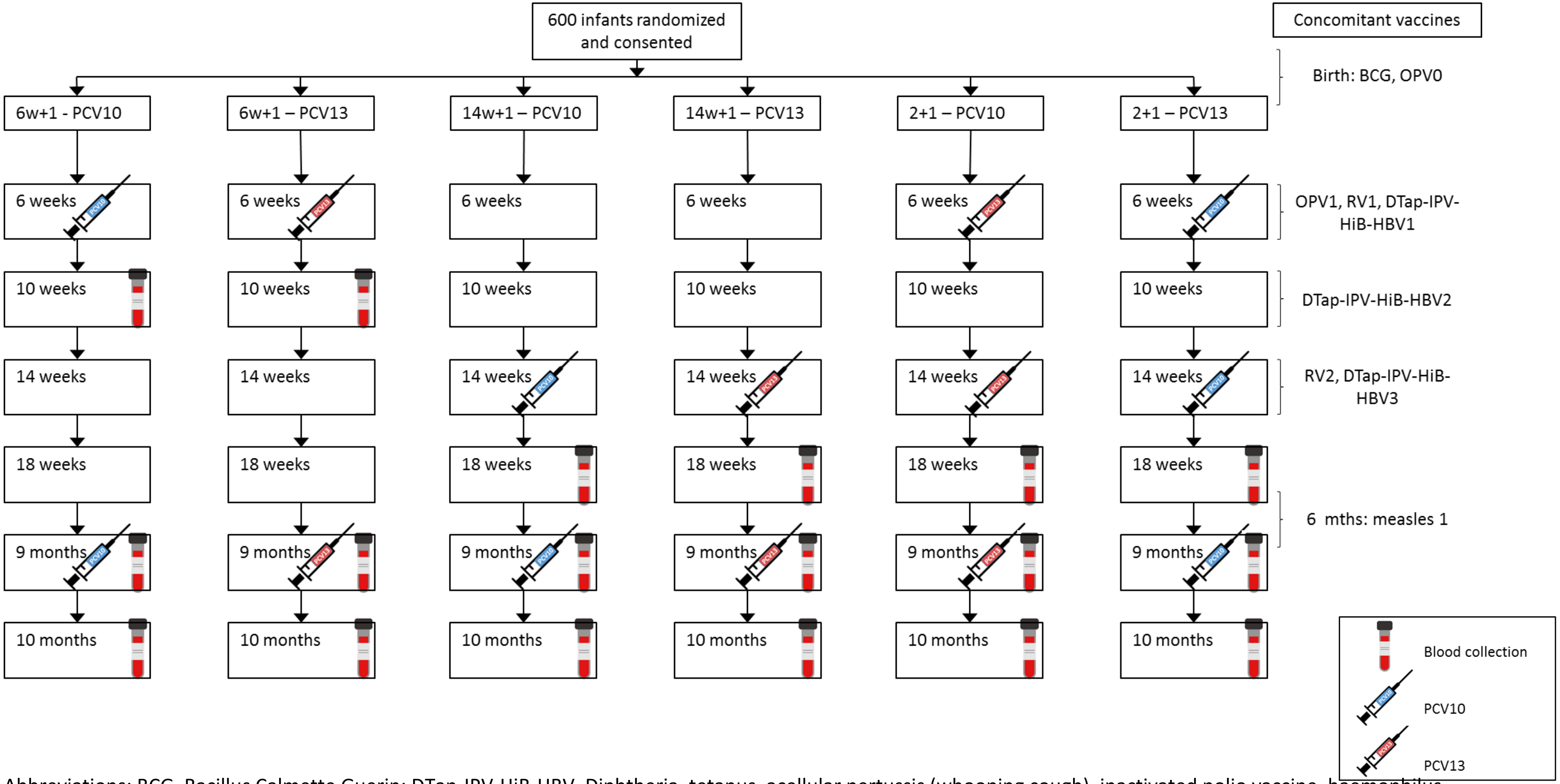

Abbreviations: BCG, Bacillus Calmette Guerin; DTap-IPV-HiB-HBV, Diphtheria, tetanus, acellular pertussis (whooping cough), inactivated polio vaccine, haemophilus influenzae type B, Hepatitis B virus; OPV, (Trivalent) Oral polio vaccine; RV, Rotavirus vaccine.

Supplementary Figure 2: Serotype-specific serum opsonophagocytic activity (OPA) geometric mean titers (GMT) one-month following a booster dose of 13-valent (PCV13; Figure 3a) or 10-valent (PCV10; Figure 3b) pneumococcal conjugate vaccine (PCV) in infants previously vaccinated with either a single dose at 6 (6w+1-PCV10/13) or 14 weeks (14w+1-PCV10/13) age or two dose at 6 and 14 weeks of age (2+1-PCV10/13) primary series.

Supplementary Figure 2a: PCV 13

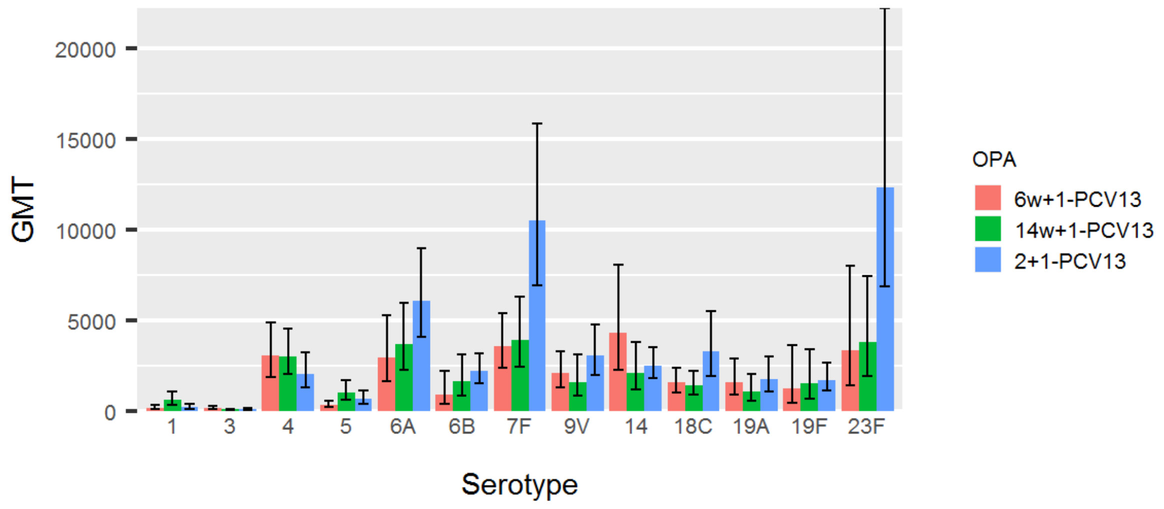

Supplementary Figure 2b: PCV10

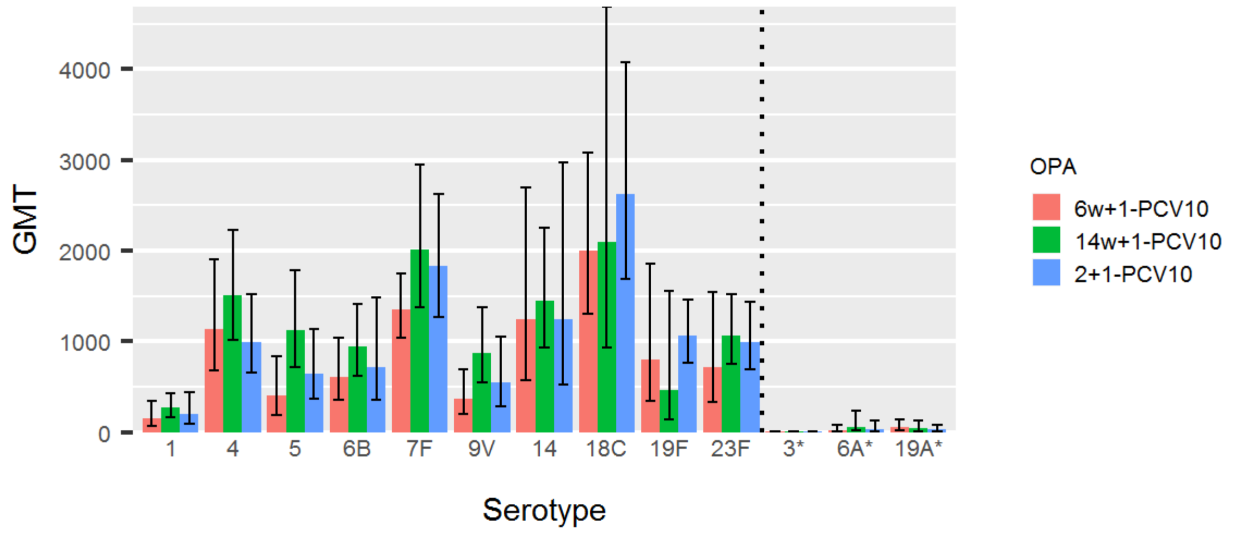

Supplementary Figure 3: Percentage with opsonophagocytic activity ≥ lower-limit-of-quantification one month post-booster dose of 13-valent (panel a) or 10-valent (panel b) pneumococcal conjugate vaccine (PCV) following a single or two dose primary-series.

Supplementary Figure 3a: PCV 13

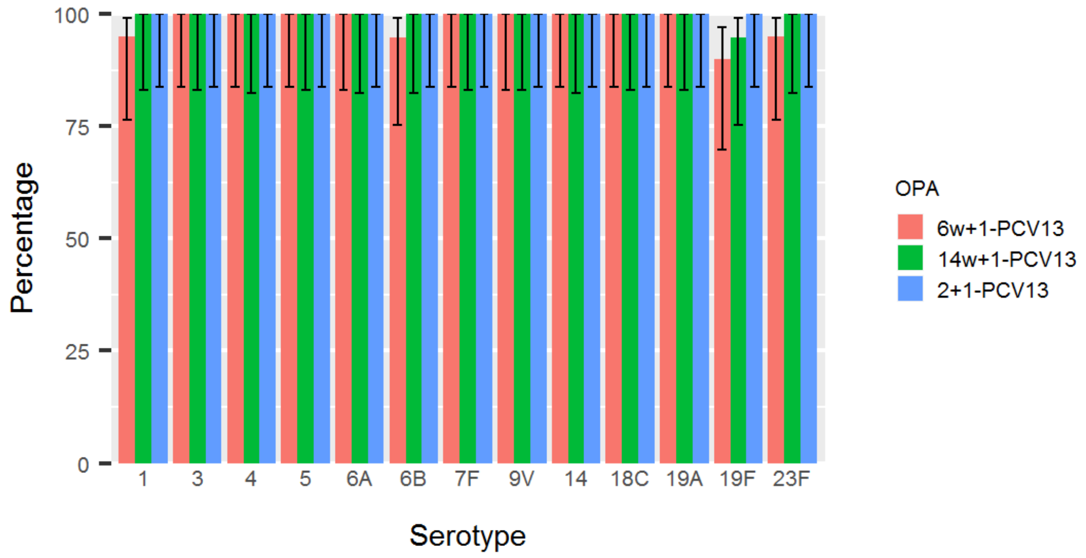

Supplementary Figure 3b: PCV 10

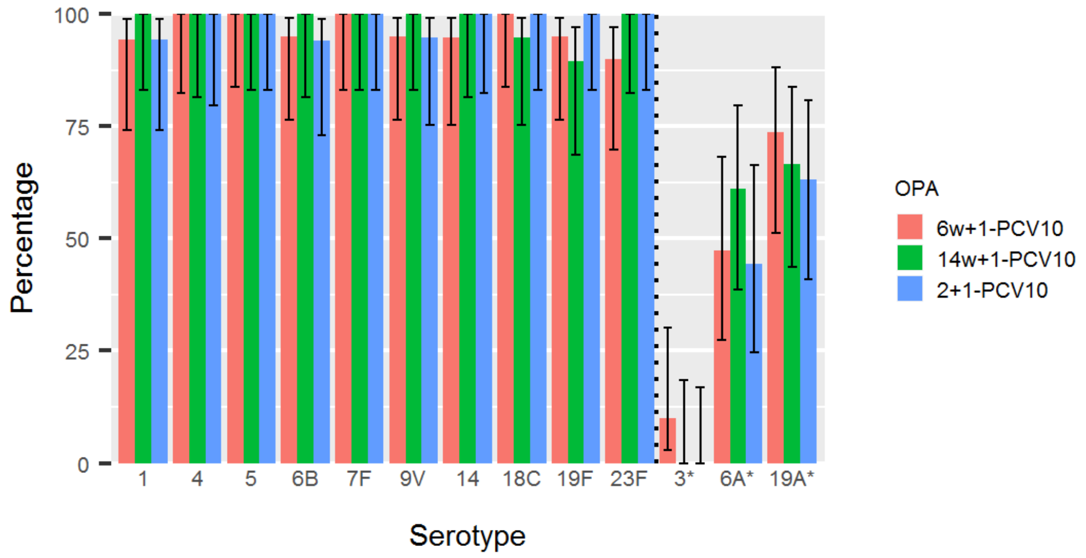

Footnote: Legend: 6 (6w+1-PCV10/13) or 14 weeks (14w+1-PCV10/13) refers to group who received a single PCV dose primary series at 6 and 14 weeks age, respectively; and 2+1-PCV refers to group who received a two-dose primary series at 6 and 14 weeks of age (2+1-PCV10/13).

*Denotes serotypes included in 13-valent but not in 10-valent pneumococcal conjugate vaccine.

The corresponding raw data is available in Supplementary Table S3.

Supplementary Figure 4: Serotype-specific IgG geometric mean antibody concentrations one-month following vaccination with a single dose at either six or 14 weeks of age, or two-dose primary series of 13-valent (Figure 1a) or 10-valent (Figure 1b) pneumococcal conjugate vaccine (PCV).

Supplementary Figure 4a: PCV13

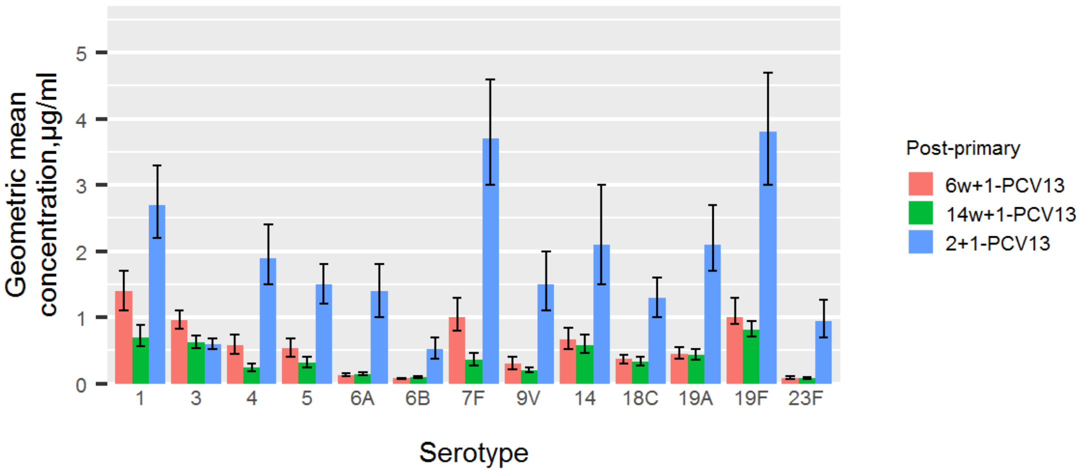

Supplementary Figure 4b: PCV10

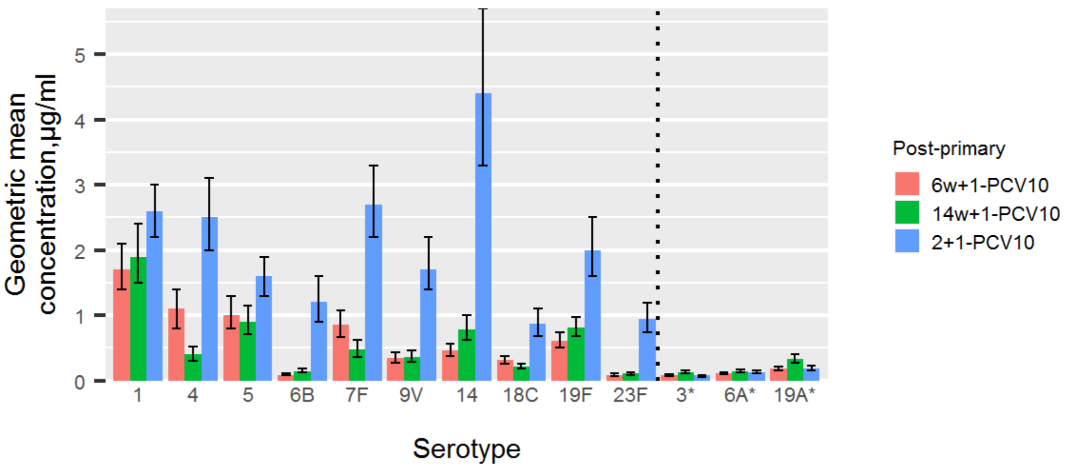

Supplementary Figure 5: Percentage of infants with serotype-specific IgG concentrations ≥0.35 µg/ml immediately before the booster dose of 13-valent (Figure 1a) or 10-valent (Figure 1b) pneumococcal conjugate vaccine, following vaccination with a single dose at either six or 14 weeks of age, or two-dose primary series of PCV.

Supplementary Figure 5a: PCV13

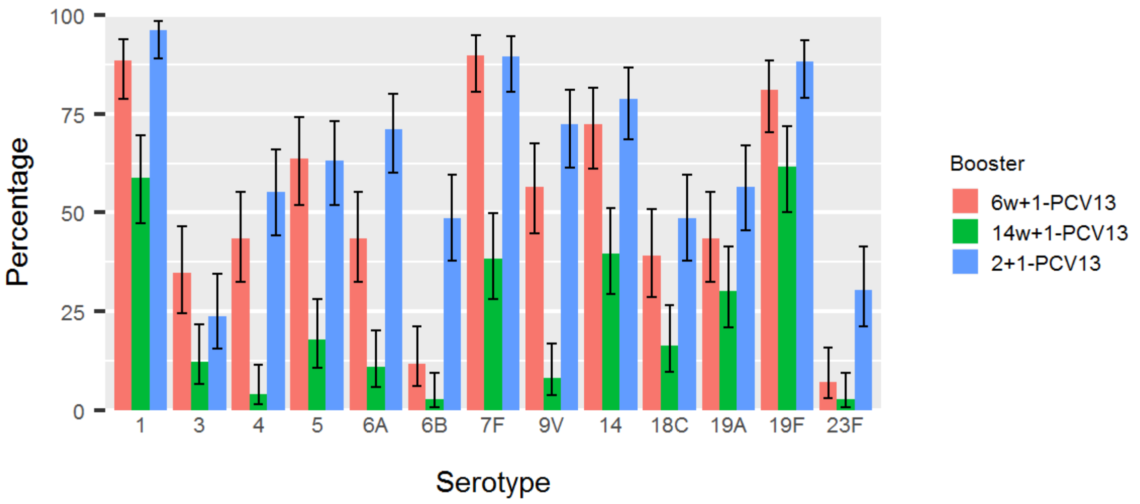

Suppl Figure 5b: PCV10

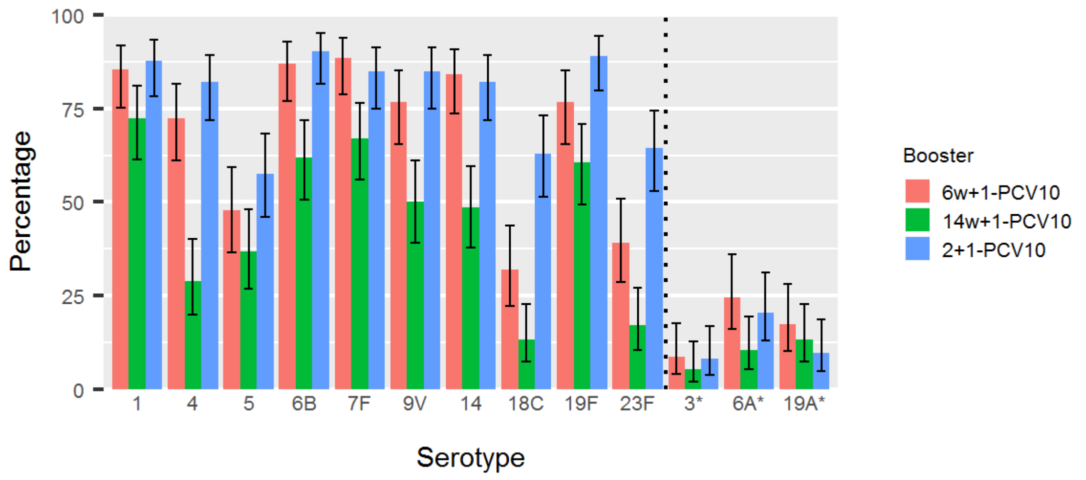

1. Andrews NJ, Waight PA, Burbidge P, et al. Serotype-specific effectiveness and correlates of protection for the 13-valent pneumococcal conjugate vaccine: a postlicensure indirect cohort study. *Lancet Infect Dis.* 2014;14(9):839-846.
2. Olwagen CP, Adrian PV, Madhi SA. Comparison of traditional culture and molecular qPCR for detection of simultaneous carriage of multiple pneumococcal serotypes in African children. *Sci Rep.* 2017;7(1):4628.
